# Understanding disorder and linker deficiency in porphyrinic zirconium-based metal–organic frameworks by resolving the Zr_8_O_6_ cluster conundrum in PCN-221

**DOI:** 10.1038/s41467-021-23348-w

**Published:** 2021-05-25

**Authors:** Charlotte Koschnick, Robert Stäglich, Tanja Scholz, Maxwell W. Terban, Alberto von Mankowski, Gökcen Savasci, Florian Binder, Alexander Schökel, Martin Etter, Jürgen Nuss, Renée Siegel, Luzia S. Germann, Christian Ochsenfeld, Robert E. Dinnebier, Jürgen Senker, Bettina V. Lotsch

**Affiliations:** 1 Max Planck Institute for Solid State Research, Heisenbergstraße 1, Stuttgart, 70569 Germany; 2 Department of Chemistry, University of Munich, Butenandtstraße 5-13, Munich, 81377 Germany; 3 e-conversion, Lichtenbergstraße 4a, Garching, 85748 Germany; 4 Center for Nanoscience, Schellingstraße 4, Munich, 80799 Germany; 5 Department of Inorganic Chemistry, University of Bayreuth, Universitätsstraße 30, Bayreuth, 95447 Germany; 6 North Bavarian NMR Center, Universitätsstraße 30, Bayreuth, 95447 Germany; 7 Deutsches Elektronen-Synchrotron (DESY), Notkestraße 85, Hamburg, 22607 Germany; 8 Present Address: Department of Chemistry, McGill University, 801 Sherbrooke St. W., Montreal, H3A 0B8 QC Canada

**Keywords:** Organometallic chemistry, Metal-organic frameworks

## Abstract

Porphyrin-based metal–organic frameworks (MOFs), exemplified by MOF-525, PCN-221, and PCN-224, are promising systems for catalysis, optoelectronics, and solar energy conversion. However, subtle differences between synthetic protocols for these three MOFs give rise to vast discrepancies in purported product outcomes and description of framework topologies. Here, based on a comprehensive synthetic and structural analysis spanning local and long-range length scales, we show that PCN-221 consists of Zr_6_O_4_(OH)_4_ clusters in four distinct orientations within the unit cell, rather than Zr_8_O_6_ clusters as originally published, and linker vacancies at levels of around 50%, which may form in a locally correlated manner. We propose disordered PCN-224 (*d*PCN-224) as a unified model to understand PCN-221, MOF-525, and PCN-224 by varying the degree of orientational cluster disorder, linker conformation and vacancies, and cluster–linker binding. Our work thus introduces a new perspective on network topology and disorder in Zr-MOFs and pinpoints the structural variables that direct their functional properties.

## Abstract (web summary)

Zirconium-based metal–organic frameworks have defective structures that are useful in catalysis and gas storage. Here, the authors study the interplay between cluster disorder and linker vacancies in PCN-221 and propose a new structure model with tilted Zr_6_O_4_(OH)_4_ clusters rather than Zr_8_O_6_ clusters.

## Introduction

Metal–organic frameworks (MOFs) have rapidly emerged as promising materials for diverse applications, such as gas storage and separation, catalysis, sensing, and biomedical applications^1^. The judicious choice and combination of organic linker and inorganic metal (cluster) allows for the precise tuning of pore shapes, sizes, and functionality toward specific applications^2–4^. Recently, MOFs based on Zr-oxo-cluster secondary building units (SBUs) linked by aromatic carboxylic acid linkers have garnered significant attention, as the high valence state of Zr^4+^ and the inertness of the Zr–O bond tolerate pronounced Zr^4+^–O bond polarization and give rise to superior stability^1,5,6^. Furthermore, a wide variety of isolated Zr-oxo-clusters, including Zr_3_, Zr_4_, Zr_5_, Zr_6_, Zr_8_, Zr_10_, and Zr_18_, have been reported, providing the basis for a rich diversity of new Zr-MOFs with different network topologies and properties^5,6^. The prototypical Zr_6_O_4_(OH)_4_ cluster is encountered most commonly and has been identified to form highly stable SBUs^5^. Well-studied MOFs built from this cluster include those with terephthalic acid (UiO-66^7^) and pyrene based (NU-1000^8^) linkers, among others^7,9–11^. Combination of Zr SBUs with the chromophore tetrakis(4-carboxyphenyl)porphyrin (TCPP) gives rise to a series of porphyrinic MOFs^12^ with interesting (opto)electronic properties, which are exploited in energy conversion schemes^13,14^ or catalysis^15^. So far, six MOFs built from Zr_6_O_4_(OH)_4_ clusters with different coordination of the TCPP linker have been reported: PCN-223^16^ and MOF-525^17^ (both 12-connected), MOF-545/PCN-222^17,18^, PCN-225^19^, and NU-902^20^ (all 8-connected), and PCN-224^21^ (6-connected).

**Fig. 1:**
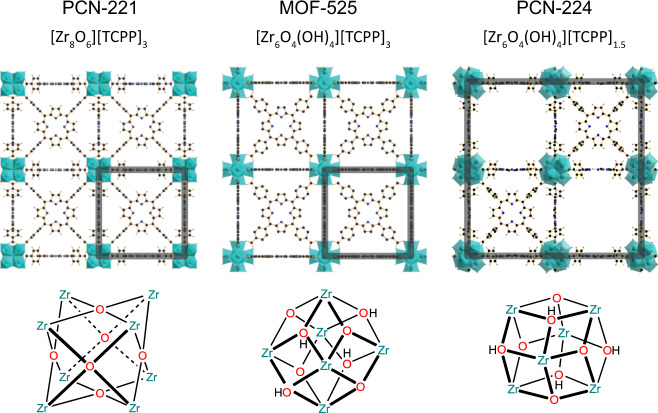
Published crystal structures of the cubic porphyrinic MOFs. PCN-221, MOF-525, and PCN-224 built of tetrakis(4-carboxyphenyl)porphyrin (TCPP) linkers and Zr-cluster (teal) with unit cells highlighted, viewed along the *c*-axis, and their corresponding Zr_8_O_6_, straight Zr_6_O_4_(OH)_4_, and tilted Zr_6_O_4_(OH)_4_ cluster, respectively^17,21,22^.

In 2013, PCN-221, a MOF similar to MOF-525, was reported featuring Zr_8_O_6_ clusters instead of Zr_6_O_4_(OH)_4_ clusters (Fig. 1)^22^. Curiously, the Zr–Zr distance in PCN-221 is only 2.69 Å, much shorter than in all inorganic Zr compounds compiled from the Inorganic Crystal Structure Database and Zr-cluster-based frameworks from the Cambridge Crystallographic Data Centre (Fig. S14). Both MOF-525 and PCN-221 are described with a cubic structure of similar unit cell size and with 12 coordination sites on the SBU. In MOF-525, the reported structure exhibits a completely planar TCPP linker, whereas in PCN-221 the phenyl rings are rotated out-of-plane by 90°^17,22^.

A difficulty in studying porphyrin-based MOF structures is the synthesis of phase-pure products, due to similar reaction conditions for all seven structures^23^. High-throughput syntheses of MOF-525 have shown the impact of modulator concentration, temperature, and reaction time on the crystallinity and phase purity, demonstrating the complexity in obtaining optimum reaction conditions^24,25^. Although not explicitly stated in the literature, it is discussed in the MOF community that syntheses of porphyrin-based Zr-MOFs are notoriously difficult to reproduce, leading to the co-existence of different MOF-525 and PCN-221 protocols^17,22,24–27^. In fact, many studies report the same product for different experimental data^21,28–41^, suggesting that the synthetic protocols and the resulting structures are still not well understood. This is exacerbated by poor quality and insufficient powder X-ray diffraction (PXRD) data making structure assignment ambiguous. As a consequence, many publications do not reveal differences in the PXRD patterns between MOF-525, PCN-221, and the ordered defect structure PCN-224^21,24–44^. However, linker vacancies in MOFs have been found to impact the material’s properties due to larger pores (adsorption applications) or open metal sites (e.g., Lewis acid catalyzed reactions), highlighting the importance of being able to reliably distinguish between these MOFs^5,45^.

Zr_6_O_4_(OH)_4_ cluster-based MOFs have been found to be remarkably tolerant toward high linker deficiencies, especially in UiO-66 and its analogs^46–50^. The tolerance is a result of large linker coordination numbers and the ability to compensate defects with either modulator molecules, hydroxy groups, or hydroxy water pairs^6,51,52^. The amount of linker vacancies can be tuned synthetically, and the inclusion of defects has been shown to proceed in a correlated manner^47,53,54^. These defect sites can be utilized to introduce functional groups by means of solvent-assisted ligand incorporation to tune the MOF’s properties^55,56^. Only recently, the notion that linker vacancies are prevalent also in the TCPP-based MOF-525 has gained attention, with vacancies reported up to 50%^25,45^.

Here, we performed a comprehensive synthetic study on PCN-221 and undertook detailed investigations of the resulting structures using total scattering pair distribution function (PDF) analysis, powder and single-crystal X-ray diffraction (PXRD/SCXRD), solid-state nuclear magnetic resonance (ssNMR) spectroscopy, and quantum chemical calculations. We have determined that PCN-221 forms with orientationally disordered Zr_6_O_4_(OH)_4_ clusters, rather than Zr_8_O_6_ cubes, correlated with vacant linker sites. The combined evidence suggests that the structure is a disordered variant of PCN-224, for which we recommend a new name, *d*PCN-224. This study thus highlights the large untapped potential for incorporating correlated orientational disorder of sub-components as a powerful design principle for developing synthetic protocols.

## Results and discussion

### Synthesis and diffraction behavior

Experimental protocols for PCN-221, MOF-525, and PCN-224 are substantially inconsistent throughout the literature. To get reliable access to these systems, we prepared a number of MOF products using different synthetic routes including the following Zr-sources: (1) zirconium(IV) chloride (MOF_ZrCl_4_) as originally reported^22^, (2) zirconyl chloride octahydrate (MOF_ZrOCl_2_ and MOF_ZrOCl_2__(II)), and (3) pre-synthesized Zr_6_O_4_(OH)_4_Bz_12_ clusters (MOF_Zr_6_). In particular, syntheses based on ZrOCl_2_ (2) showed good reproducibility (details on all syntheses see Supplementary Information “Synthetic procedures”). In addition, PCN-224 with TCPP linker vacancies appearing in an ordered 3D checkerboard fashion (Fig. 1) was synthesized as reference.

**Fig. 2:**
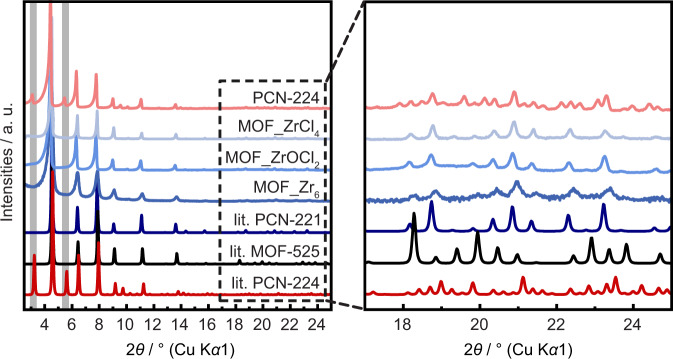
Powder X-ray diffraction (PXRD) patterns for samples and models. Comparison of measured PXRD patterns (Cu K*α*_1_) of MOF_ZrCl_4_, MOF_ZrOCl_2_, MOF_Zr_6_, and experimental PCN-224 and simulated PXRD patterns (lit.) of PCN-221, MOF-525, and PCN-224^17,21,22^. The gray bars highlight the superstructure reflections of PCN-224 at 3.2 and 5.5° 2*θ*.

The PXRD patterns of the products MOF_ZrCl_4_, MOF_ZrOCl_2_, and MOF_Zr_6_ are all very similar and do not show features that would suggest significantly different structures (Fig. 2). We compared these to calculated PXRD patterns based on the published crystal structures of PCN-221, which is reported with a Zr_8_O_6_ cluster and twisted phenyl linker conformation, and MOF-525, reported with a Zr_6_O_4_(OH)_4_ cluster and planar linker conformation (for structural details see Fig. 1). At low scattering angles the simulated patterns of the published structure of PCN-221 and MOF-525 are strikingly similar, due to the identical space group $Pm\bar 3m$ and similar lattice parameters (19.51 and 19.39 Å, respectively)^17,22^. This makes structure assignment difficult in the absence of good quality data extending to higher angles. Nevertheless, all sample patterns show better agreement with the simulated structure of PCN-221, including the MOF_Zr_6_ sample synthesized from preformed Zr_6_O_4_(OH)_4_Bz_12_ clusters. This is surprising, since PCN-221 is published with Zr_8_O_6_ clusters. A rearrangement of the pre-synthesized Zr_6_O_4_(OH)_4_ cluster is not expected during the synthesis and crystallization of MOF_Zr_6_, raising the question of whether Zr_6_O_4_(OH)_4_ or Zr_8_O_6_ clusters are actually present. For comparison, we measured PXRD data of experimental PCN-224, with ordered TCPP linker vacancies, showing superstructure reflections at 3.2 and 5.5° 2*θ* (Fig. 2).

### Cluster structure

**Fig. 3:**
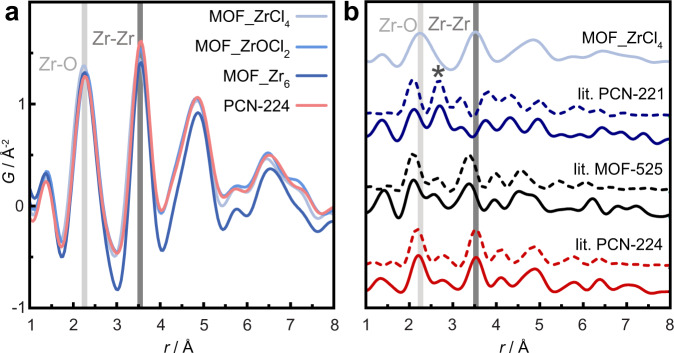
Local structures of MOF samples and models. **a** Experimental pair distribution functions (PDFs) of MOF_ZrCl_4_, MOF_ZrOCl_2_, MOF_Zr_6_, and experimental PCN-224; **b** experimental PDF of MOF_ZrCl_4_ compared to the simulated PDFs (lit.) of the published MOF structures (solid line) and SBUs only (dashed line) of PCN-224, MOF-525, and PCN-221^17,21,22^. The star highlights the Zr–Zr distance of 2.69 Å in PCN-221.

Due to the ambiguity in cluster constitution, based on comparison of measured and calculated PXRD data, we proceeded to use total scattering PDF analysis to investigate the short-range order of the Zr-oxo cluster. For all samples, the PDF data show peaks at the same atom-pair distances, which corroborates similar local Zr environments within the clusters of different samples (Fig. 3a). The experimentally determined Zr–O distances of 2.28 Å and Zr–Zr distances of 3.54 Å are found to match with those expected for a Zr_6_O_4_(OH)_4_ cluster, rather than 2.12 and 2.69 Å, respectively, for the Zr_8_O_6_ cluster (Fig. 3b)^22^. The reported structure for MOF-525 gives slightly contracted distances, Zr–O (2.06 Å) and Zr–Zr (3.32 Å) compared to the experimental data, while the structure of PCN-224, with Zr–O (2.15 Å) and Zr–Zr (3.50 Å), gives the best overall agreement to the local structure. These findings are in agreement with quantum chemical calculations on the structure of a Zr_8_O_6_ cluster, for which no energetically favorable geometry could be successfully determined. In contrast, an optimized Zr_6_O_4_(OH)_4_ cluster shows very good agreement with the Zr–Zr distances from the PDF refinement (Figs. S19–S21 and S33).

### Structure model from X-ray data

**Fig. 4:**
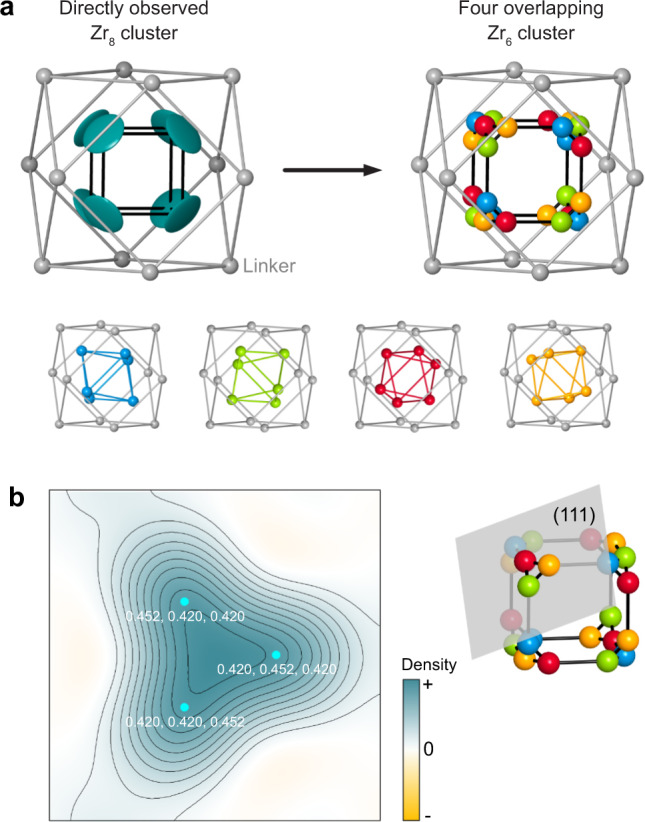
Cluster orientations from single-crystal X-ray diffraction (SCXRD). **a** Zr_8_ cluster (teal) determined from SCXRD data, with a flat disk-shaped ellipsoid of the Zr atom (left) and a truncated cube created by the overlap of four different orientations of Zr_6_O_4_(OH)_4_ clusters (blue, green, red, and orange), each occupied by 25% (right); **b** Fourier map of the (111) plane through the truncated corners of the cube showing the electron density map of three Zr atoms (positions indicated in white).

All three synthetic routes MOF_ZrCl_4_, MOF_ZrOCl_2_, and MOF_Zr_6_ yielded a MOF whose structure agrees well with the published structure for PCN-221 with a Zr_8_O_6_ cluster in the long range (PXRD), but locally contradicts the presence of Zr_8_O_6_ clusters. To elucidate this discrepancy, we studied the structure of the MOF by SCXRD. Several reaction parameters had to be adjusted to grow single crystals of the same MOF with suitable sizes for SCXRD (Fig. S22). Indexing revealed a primitive cubic unit cell with *a* = 19.315(2) Å, and the initial structure solution gave a Zr_8_O_6_ cluster similar to the structure of PCN-221^22^. However, residual electron density arranged in a triangular shape remained around each Zr atom, manifesting itself as extremely flat, disk-shaped thermal ellipsoids of the Zr in the Zr_8_O_6_ cluster (Fig. 4a), as previously reported^22^. The corners of the Zr_8_ cube point toward all eight triangles of the cuboctahedral cage formed by the carbon atoms of the coordinating linker molecules (Fig. 4). In fact, it is possible to describe such a distribution of electron density by superimposing four different Zr_6_ cluster orientations as seen in the 2 × 2 × 2 supercell of PCN-224, so that the corners of any Zr_6_ octahedron point to six of the eight cuboctahedron’s triangles. The four orientations are related by 90° rotations around the crystallographic axes (Fig. 4a). Overlapping these four Zr_6_ clusters adds up to a truncated cube (corner-to-corner distance = 2.70 Å) with a strong resemblance to the Zr_8_O_6_ cluster structure published for PCN-221 (Zr–Zr = 2.69 Å). Using this disoriented model, the *R* value was improved by 12%, compared to the structure model built of Zr_8_O_6_ cubes. For more details on the SC refinement and crystallographic data please refer to the Supplementary Information (“Single-crystal X-ray diffraction”). The crystal structure file is provided in Supplementary Data 1. Our analysis is further consistent with the PDF data that suggest the local environment to be more similar to PCN-224. We found additional evidence for our structural model in the Fourier map of the (111) crystallographic plane through the truncated corners of the cube that show a three-fold symmetry in the electron density map with maxima on the superimposed Zr sites (Fig. 4b).

The tilted Zr_6_O_8_ cluster orientations offer 12 simultaneous carboxylate positions coordinating such that six of them bridge two adjacent Zr atoms (bridging ligands) and the other six chelate single Zr atoms (chelating ligands). This building unit shows similarities to the hexagonal TCPP-based PCN-223^16^ featuring both bridging and chelating ligands, rather than only bridging as in MOF-525 and PCN-224.

The SCXRD model was solved with a 90° dihedral angle between the porphyrin and the phenyl ring, rather than 0° as reported for MOF-525. The refinement of this angle is prohibited by the symmetry restrictions of the cubic space group $Pm\bar 3m$. These values are closer to the published structure of PCN-224, in which the phenyl rings of the porphyrin linker are twisted out-of-plane by ~78°^21^. The potential energy surface obtained from quantum chemical calculations of TCPP in the gas phase shows a steep maximum at 0° and a very shallow minimum in the range of 60–90° (Fig. S36 and Table S15). This confirms the preference for a twisted dihedral angle of the phenyl rings.

The occupancy of the linker was further investigated by SCXRD, PDF, Rietveld, and chemical analysis (Supplementary Information “Chemical composition analysis”) as well as ssNMR spectroscopy. SCXRD analysis revealed that the porphyrin moiety of the linker is not fully occupied, but rather ~50%; therefore, only six out of the 12 sites on the Zr_6_O_8_ cluster are coordinated by TCPP, similar to PCN-224. Compared to the porphyrin moiety, the phenyl rings refined to a higher occupancy of ~75%. This suggests that besides six TCPP linkers, leftover pore content may occupy the TCPP-vacant sites.

**Fig. 5:**
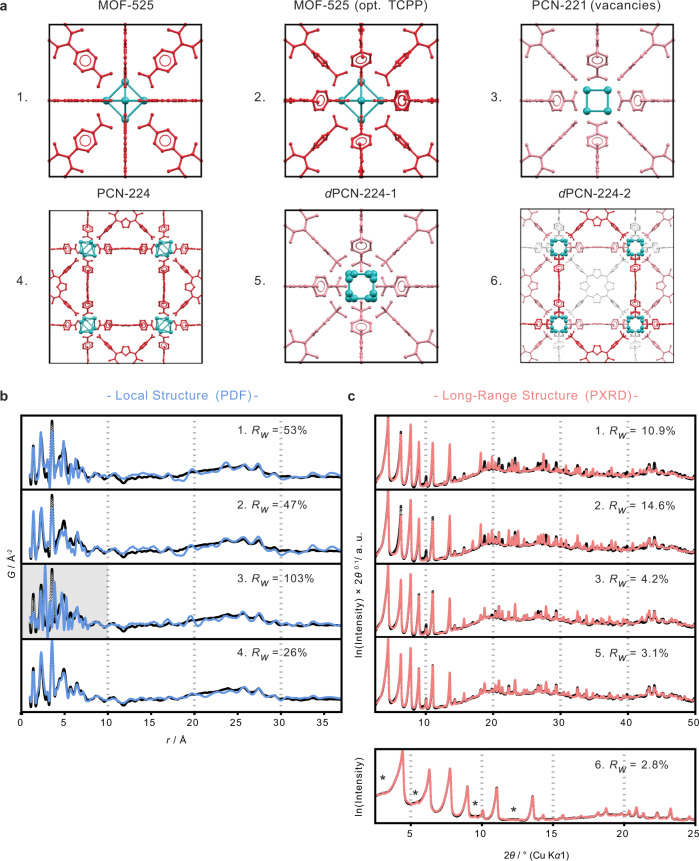
Structure models with corresponding pair distribution function (PDF) and Rietveld refinements. **a** MOF structures shown with Zr (teal) and tetrakis(4-carboxyphenyl)porphyrin (TCPP) linkers with 100% (red), 50% (pink), and 0% (gray) occupancy: (1) MOF-525 with planar TCPP linkers and no vacancies, (2) MOF-525 structure with twisted phenyl rings in the TCPP linker and no linker vacancies, (3) PCN-221 (Zr_8_ clusters) with equally distributed linker vacancies, (4) PCN-224 structure with ordered TCPP vacancies, (5) *d*PCN-224-1 built of Zr_6_ clusters with four different orientations and equally distributed linker vacancies, and (6) *d*PCN-224-2 with relative cluster orientations and linker occupation refined separately for each individual linker and cluster site, allowing for different possible local environments (the linker colors of (6) are an artistic representation and not specific to occupancies of the refined model). **b** PDF fits compared to experimental *d*PCN-224 (blue, PDF of MOF_ZrOCl_2_ measured using synchrotron radiation), showing poor agreement between the local environments in structures 1–3 and good agreement with the local structure of PCN-224 (structure 4). **c** Improvement of the Rietveld refinements of experimental *d*PCN-224 (pink, also sample MOF_ZrOCl_2_) for models shown. The extra intensities generated by the 2 × 2 × 2 supercell model of *d*PCN-224 are highlighted (*). *Rw* is the residual value (see Supplementary Information “Pair distribution function analysis”).

PDF analysis provided further insights into the linker vacancies and cluster orientations (Fig. 5). The intermediate-range atomic density distribution from 5–40 Å suggests a high degree of similarity to experimental PCN-224 with respect to the preferred coordination of linkers around the Zr-oxo clusters, and the relative concentration of linker present, confirmed by comparison to published models (Fig. 5b). The PDFs of all samples thus also indicate 50–60% porphyrin occupancy. Furthermore, slightly increased broadening of features at long distances suggest a more disordered long-range structure compared to the PCN-224 sample (Fig. S17). This could be attributed to more randomly distributed vacancies, in contrast to ordered vacancies in PCN-224.

For Rietveld refinements (Fig. S6), accounting for orientationally averaged clusters, nonplanar porphyrin linkers, and random linker vacancies led to substantially improved agreement with the PXRD data. Additional electron density coordinating cluster surface sites was required to obtain a good fit. This could be primarily accounted for by remaining water content, though contribution from other remaining coordinative species is possible. Multiphase refinements performed found no preference for any ordered domains of MOF-525-like structure.

The models discussed still could not explain weak, diffuse features consistently observed at the superstructure reflection positions of PCN-224 (Figs. 5c and S9), which were also observed by SCXRD (Fig. S24). We built a 2 × 2 × 2 supercell and refined separate occupancy factors for each individual linker and cluster orientations for each cluster site, allowing for different local vacancy–orientation environments. This gave a good fit over the entire angular range and reproduced the weak features; however, it is not sufficient to identify a distinct local structure patterning of orientations and vacancies, as other supercell models can also produce similar diffuse features. Altogether, the findings suggest that these structures form more as a frustrated network than as a simple crystal, though the average structure can be described by orientationally disordered Zr_6_O_4_(OH)_4_ clusters and linker vacancies distributed more randomly than in PCN-224. As such, we propose disordered PCN-224 (*d*PCN-224) as a more systematic description of PCN-221. The crystal structure models from refinement using a single unit cell and with a 2 × 2 × 2 supercell, as presented in Fig. 5a models 5 and 6, are given in Supplementary Data 2 and 3, respectively.

### Local environment of the cluster

**Fig. 6:**
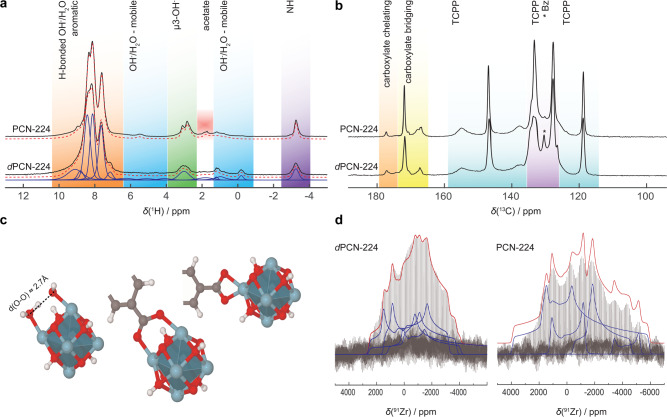
Solid-state nuclear magnetic resonance (ssNMR) spectroscopic analysis of *d*PCN-224 (MOF_ZrOCl_2_) and PCN-224. **a** ^1^H high-resolution magic angle spinning (MAS) NMR spectra recorded at a field of 23.48 T (ν_0_(^1^H) = 1 GHz). Characteristic resonances for the aromatic and NH protons of the tetrakis(4-carboxyphenyl)porphyrin (TCPP) linker, the µ3-OH groups of the Zr_6_O_4_(OH)_4_ cluster^57^, hydroxy groups, and water molecules^51,52^ are highlighted by color-coded regions. **b** ^13^C cross polarization magic angle spinning (CPMAS) spectra with assignment of the aromatic carbons of the TCPP linkers and benzoic acid (Bz), and chemically inequivalent carboxylic acid groups, characteristic of chelating and bridging binding motifs for the carboxylic acid groups to the cluster. **c** Main coordination motifs for the Zr_6_O_4_(OH)_4_ clusters, from left to right: hydroxy water pairs and bridging and chelating carboxylate groups, which were found in decreasing proportions along this sequence. **d** ^91^Zr quadrupolar Carr-Purcell-Meiboom-Gill variable offset cumulative spectroscopy (qCPMG VOCS)^62–65^ consisting of at least four different quadrupolar lineshapes (blue), typical for quadrupolar coupling constants between 20 and 25 MHz. Together with the non-axial symmetry of the quadrupolar coupling tensors, an environment with a low symmetry and complex coordination of the Zr atoms is revealed within the cluster. Individual lineshapes and spectral fits are illustrated in blue and red, respectively.

To obtain insights into the local coordination of the Zr-oxo cluster in PCN-221 (*d*PCN-224), a comprehensive set of solid-state NMR measurements was carried out. High-resolution ^1^H, ^13^C, and ^15^N magic angle spinning (MAS) NMR spectra of *d*PCN-224 and PCN-224 (Figs. 6a, b and S25–S27) are very similar, further supporting the proposed structural relation between both compounds on local length scales. The NMR spectra show well-resolved resonances for all characteristic chemical groups of the TCPP linkers, the modulator molecules (acetate and benzoate (Bz)), and the Zr clusters. In particular, the presence of sharp and well-defined resonances for the µ3-OH groups at around 3.3 ppm in the ^1^H MAS NMR spectra (Figs. 6a and S25) underlines the presence of Zr_6_O_4_(OH)_4_ units^57^. The ratio between the intensities of the µ3-OH (≈3.3 ppm) and the NH (≈–3.2 ppm) resonances allowed us to derive a TCPP linker occupancy of 53(4)% for *d*PCN-224 and 40(4)% for PCN-224 of the Zr_6_O_4_(OH)_4_ cluster.

The aromatic regions (7–9 ppm) of the ^1^H NMR spectra show significantly more intensity than expected for TCPP linkers alone. Combined with the small amount of modulator molecules, as determined from the ^1^H and ^13^C cross polarized magic angle spinning spectra (Figs. 6a and S25 and S26), we conclude that the remaining six (*d*PCN-224) to seven (PCN-224) coordinatively unsaturated sites at the Zr_6_O_4_(OH)_4_ clusters are mainly saturated by hydroxy water pairs (for details see Supplementary Information “Solid-state NMR spectroscopy”). A similar situation was recently reported for the same cluster type in NU-1000^51,58^. The strong downfield shift for the protons within such pairs translates into comparably short O–O distances between 2.6 and 2.7 Å (Fig. 6), characteristic for strong hydrogen bonds^52,58,59^. The additional spectral intensity between 6 and 4 ppm was attributed to mobile water in the neighborhood of the Zr clusters. Both the hydroxy water pairs at the coordinatively unsaturated sites of the clusters, as well as the mobile water may account for the additional electron density close to the cluster surface sites, which is required for the Rietveld refinement of the PXRD data.

The carboxylic region of the ^13^C MAS NMR spectra for *d*PCN-224 and PCN-224 contains several signals between 167.3 and 177.2 ppm centered around a main peak at ≈171.9 ppm (Fig. 6b). Such a broad spread reflects different binding modes between the Zr_6_O_4_(OH)_4_ clusters and the carboxylate units of the TCPP linkers and residual modulator molecules, in combination with a configurational variance from cluster to cluster. Based on DFT calculations (Fig. S30 and ref. ^60^) we assigned the central resonance and its high-field region (165–173 ppm) to carboxylates in a bridging motif and the low-field region (173–176 ppm) to a chelating motif. As such, the primary coordination motif consists in bridging TCPP linkers for both *d*PCN-224 and PCN-224, in accordance with the published structure model of PCN-224^21^ and reported calculations showing that bridging carboxylate groups are favorable over chelating ones^60^. A small part of carboxylate groups is found in a chelating motif reflecting the local disorder of the clusters in both *d*PCN-224 and PCN-224.

The distribution of different binding modes is also reflected in the lineshapes of the ^91^Zr wideline NMR spectra (Fig. 6d). They consist of at least four different quadrupolar shapes with non-axial symmetric coupling tensors and coupling constants (C_Q_) between 10 and 25 MHz. This proves a markedly more diverse coordination for the individual Zr atoms compared to a free Zr_6_O_4_(OH)_4_Bz_12_ cluster with 12-fold Bz coordination^61^ (Fig. S31). Compared to the free cluster, the broader variance for *d*PCN-224 and PCN-224 reflects the disorder induced by attached hydroxy water pairs, residual modulator molecules, and the strain on the Zr clusters imposed by the framework.

### Linker vacancies

We revisit the implications of linker vacancies at ~50% occupation as consistently derived from SCXRD, Rietveld, PDF, and ssNMR. Recently, highly defective MOF-525 structures with up to 50% linker vacancies were reported, further supporting the observation of high vacancy concentrations in our proposed structure of PCN-221 (*d*PCN-224)^25,45^. The favorable coordination of bridging ligands^60^, as implied by ssNMR, suggests that TCPP vacancies should preferentially occur where chelating coordination would otherwise be required, similar to the reported PCN-224 structure. The presence of diffuse superstructure features in both PXRD and SCXRD further suggest that small domains of vacancy ordering may occur. Both *d*PCN-224 and MOF-525 are built from Zr_6_ clusters, however, MOF-525 only has one cluster orientation, where the primary axes of the octahedron are aligned with the unit cell axes. This straight orientation imposes a more energetically demanding conformation of the TCPP molecules with either fully planar TCPP molecules, or with the carboxylate groups oriented unfavorably perpendicular to the twisted phenyl groups (Fig. S35 and S36). Unlike the MOF-525 models, the six bridging TCPP linkers in *d*PCN-224 both have twisted phenyl rings and coplanar carboxylate groups, which represents an energetically more favorable situation. We thus conjecture that the formation of MOF-525 with only straight cluster orientations may be hindered by a combination of kinetic limitations and configurational entropy considerations. If TCPP initially attaches to one of the six discussed bridging sites between differently tilted clusters, the 12-fold bridging-only coordination of MOF-525 can no longer form. The bridging sites with respect to the tilted cluster are shown in Fig. S37. This may well explain why we consistently obtained *d*PCN-224 as the dominant structure. While we cannot exclude the possibility of site-isolated MOF-525-like cluster orientations in *d*PCN-224, possibly in regions of higher coordination, we found no evidence for the formation of ordered domains of the reported MOF-525 structure.

In conclusion, we have resolved the long-standing question regarding the formation of a Zr_8_O_6_ SBU within the MOF PCN-221. Based on a combination of local and long-range sensitive structural techniques, we have revealed that the superposition of four statically disordered Zr_6_O_4_(OH)_4_ clusters appears on average as a truncated cube, which can easily be misinterpreted as a Zr_8_O_6_ cluster. The orientations of the clusters, linker vacancies up to 50%, and the conformation of the TCPP linker are all reminiscent of PCN-224; however, PCN-224 is distinct in that it forms an ordered 3D checkerboard arrangement of linker vacancies and ordered relative cluster orientations. We therefore recommend to describe the structure of PCN-221 as a disordered variant of PCN-224: *d*PCN-224. The remaining coordination sites at the Zr_6_O_4_(OH)_4_ clusters are completed by hydroxy water pairs and modulator molecules. The origin of the linker vacancies was traced back to two different coordination environments in PCN-221 (*d*PCN-224)—bridging and chelating. While the former is energetically more favorable according to quantum chemical calculations, the relatively flat energy landscape for the different coordination modes and linker conformations, and the high configurational entropy associated with the 12 coordination sites of the Zr_6_O_4_(OH)_4_ clusters rationalizes the defect-prone nature of these MOFs. This work thus sheds light on the interplay between network topology, defects, and disorder in Zr-MOFs, and opens up a unique design space by invoking additional degrees of freedom such as correlated cluster orientation, cluster disorder, and cluster–linker binding modes. In the context of these parameters, it may be useful to look for similar phenomena in other MOF systems displaying ordered defect variants and investigate the corresponding differences in properties. Once controlled, these design criteria could unleash a huge untapped potential for directing the functional properties of MOFs.

## Supplementary information

Supplementary Information

Supplementary Data 1

Supplementary Data 2

Supplementary Data 3

Description of Additional Supplementary Files

## Data Availability

The data supporting the findings of this study are presented within the article and its Supplementary Information files. Datasets are available from the corresponding authors upon request.
