# Supplementary material for: Understanding disorder and linker deficiency in porphyrinic zirconium-based metal–organic frameworks by resolving the Zr8O6 cluster conundrum in PCN-221
Source: Nat Commun. 2021 May 25;12:3099. doi: 10.1038/s41467-021-23348-w (PMC8149457; doi:10.1038/s41467-021-23348-w)
Supplement: Supplementary file 1 — Supplementary Information [file 41467_2021_23348_MOESM1_ESM.pdf]

Supporting information

Understanding disorder and linker deficiency in porphyrinic zirconium-based metal–organic frameworks by resolving the Zr_8O_6 cluster conundrum in PCN-221

Charlotte Koschnick[1,2,3,4,9], Robert Stäglich[5,6,9], Tanja Scholz[1,9], Maxwell W. Terban[1,9], Alberto von Mankowski[1,2,3,4], Gökcen Savasci[1,2,4], Florian Binder[1,2], Alexander Schökel[7], Martin Etter[7], Jürgen Nuss[1], Renée Siegel[5,6], Luzia S. Germann[1,8], Christian Ochsenfeld[2,1,4], Robert E. Dinnebier[1], Jürgen Senker[5,6,*], Bettina V. Lotsch[1,2,3,4,*]

[1]Max Planck Institute for Solid State Research, Heisenbergstraße 1, 70569 Stuttgart, Germany
[2]Department of Chemistry, University of Munich, Butenandtstraße 5-13, 81377 Munich, Germany
[3]e-conversion, Lichtenbergstraße 4a, 85748 Garching, Germany
[4]Center for Nanoscience, Schellingstraße 4, 80799 Munich, Germany
[5]Department of Inorganic Chemistry, University of Bayreuth, Universitätsstraße 30, 95447 Bayreuth, Germany
[6]North Bavarian NMR Center, Universitätsstraße 30, 95447 Bayreuth, Germany
[7]Deutsches Elektronen-Synchrotron (DESY), Notkestraße 85, 22607 Hamburg, Germany
[8]Present Address: Department of Chemistry, McGill University, 801 Sherbrooke St. W., H3A 0B8 Montreal, QC, Canada
[9]These authors contributed equally
[*] Corresponding authors

email: b.lotsch@fkf.mpg.de; juergen.senker@uni-bayreuth.de

1. Experimental ... 3

1.1. Materials ... 3

1.2. Instruments and Methods ... 3

1.3. Synthetic procedures ... 6

2. Results and discussion ... 9

2.1. SEM Images ... 9

2.2. Powder X-Ray Diffraction ... 10

2.3. Chemical Composition Analysis ... 18

2.4. Pair distribution function analysis ... 22

2.5. Single-Crystal X-ray Diffraction ... 32

2.6. Solid-state NMR spectroscopy ... 37

2.7. Quantum chemical calculations ... 45

2.8. Coordination of tilted Zr_6 cluster in *d*PCN-224 ... 51

References ... 52

1. Experimental

1.1. Materials

Tetrakis(4-carboxyphenyl) porphyrin ($TCPPH_4$) was purchased from Tokio Chemical Industry. Zirconylchloride octahydrate and benzoic acid were purchased from Sigma Aldrich. N,N-Diethylformamide (DEF) was purchased from Alfa Aesar. N,N-Dimethylformamide (DMF) and Acetone were purchased from VWR. Acetic acid and hydrochloric acid were purchased from Carl Roth. All chemicals were used as received without further purification.

1.2. Instruments and Methods

Ultrasonication was conducted via an ELMASONIC S 100 bath equipped with a high-performance 37 kHz sandwich transducer and state-of-the-art microprocessor. An *Initiator Classic* from BIOTAGE was used for microwave assisted synthesis. Centrifugation was performed with a benchtop centrifuge *Sigma-3-30K* from SIGMA. For SEM analysis MOF suspensions were spin-coated onto silicon wafers with a *WS-650S-NPP Lite* device from LAURELL TECHNOLOGY CORPORATION.

Microscope images were taken with a CMOS camera connected to a *DM2500* light microscope from LEICA. The morphology of particles were examined with scanning electron microscopy (SEM; Vega TS 5130 MM, Tescan). SEM were performed with a secondary electron (SE) detector with a Merlin SEM (Zeiss). Particle sizes were investigated with the software ImageJ.

Powder X-Ray Diffraction

Laboratory XRPD patterns were collected at room temperature on a Stoe Stadi-P diffractometer with Cu $K\alpha_1$ radiation (λ = 1.540596 Å), a Ge(111) Johann monochromator, and a DECTRIS Mythen 1K detector in Debye-Scherrer geometry. The samples were loaded into 1 mm inner diameter polyimide capillaries and measured over a range of 2θ = 1.000–90.505°, with 0.405° step size and 27 s counting time per step. The PXRD pattern of the single crystal sample was loaded into a 0.3 mm inner diameter capillary and measured over a range of 2θ = 2.000–20.255°, with 2.61° step size and 50 s counting time per step. The software package WinXPOW was used to analyze all data obtained by powder diffractometry and to simulate theoretical diffraction patterns.

Pawley and Rietveld refinements were performed using TOPAS v6 on diffraction data collected using Cu $K\alpha_1$ radiation. All refinements were performed using a 2θ offset correction, full axial model, and Lorentzian and Gaussian strain and crystallite size broadening convolutions to correct for instrumental and

morphological peak-shape effects. The background was defined as a highly broadened Pawley phase. For Rietveld refinements of models giving a poor match to high-angle features, B_{iso} was fixed to 10.0 $Å^2$ for all atoms. For the *d*PCN-224-1 model, B_{iso} for linkers was fixed to 5.0 $Å^2$ and allowed to refine for cluster atoms. For the *d*PCN-224-2 model, both values were allowed to refine.

All models with only cluster and linkers, regardless of occupancies of any components, did not produce enough intensity to match the 100 diffraction peak. Use of the resulting Fourier map identified missing electron density on six locations around the clusters between the TCPP linkers. The missing intensities were successfully modeled using pseudo-atoms[1] (defined as Ag atoms in the crystal structure) with large atomic displacement parameters. Since all oxygen sites on the cluster were left at 100% occupancy, the additional electron density could be accounted for primarily by additionally coordinating layers of water, as seen in NMR of undried samples, though we expect that some contributions from other coordinating species is possible.

Single Crystal X-Ray Diffraction

The crystal was mounted with some high-viscous oil on a loop made of Kapton foil. Diffraction data were collected at 100 K with a D8-Venture X-ray diffractometer equipped with a Photon III detector (Bruker AXS, Karlsruhe, Germany), a microfocus X-ray source IµS 3.0 (Cu Kα radiation, Incoatec, Geesthach, Germany), and a Cryostream 800 cooling device (Oxford Cryosystems, Oxford, U.K.). The reflection intensities were integrated with the SAINT subprogram in the Bruker Suite software package.[2] A multi-scan absorption correction was applied using SADABS.[3] The structure was solved by direct methods and refined by full-matrix least-squares fitting with the SHEXTL software package.[4,5]

Pair distribution function analysis

Total scattering measurements were carried out using P02.1, the Powder Diffraction and Total Scattering Beamline, at PETRA III of the Deutsches Elektronen-Synchrotron (DESY). The rapid acquisition PDF method (RAPDF)[6] was used with a large-area 2D PerkinElmer detector (2048×2048 pixels, 200×200 µm each) and sample-to-detector distance of 335.8767 mm. The incident energy of the x-rays was 59.850 keV (λ = 0.20716 Å). Samples were loaded into 1 mm inner diameter polyimide capillaries. An empty capillary was measured as background and subtracted, and a LaB6 standard was measured at room temperature for calibration of the setup. Calibration, polarization correction, and azimuthal integration to 1D diffraction patterns were performed using the software Fit2D.[7,8]

Additional total scattering measurements were performed on the same samples in-lab using a Stoe Stadi-P diffractometer with Mo $K\alpha_1$ radiation (λ = 0.7093 Å), a Ge(111) Johann monochromator, and a DECTRIS

Mythen 1K detector in Debye-Scherrer geometry. Measurements were carried out over a range of $2\theta = 0–106.5°$, with 0.495° step size, 14 s counting time per step, summed over 19 repetitions. Data were directly corrected for the 2θ offset of the instrument.

Further correction and normalization of the 1D diffraction intensities were carried out to obtain the total scattering structure function, $F(Q)$, which was Fourier transformed to obtain the PDF, $G(r)$ using PDFgetX3 within xPDFsuite.[9,10] The maximum value used in the Fourier transform of the total scattering data was 15.55 $Å^{-1}$ for the synchrotron data and 14.2 $Å^{-1}$ for the Mo $K\alpha_1$ laboratory data.

Solid-state NMR spectroscopy

Solid-state NMR studies of ^1H were performed on Bruker Avance III HD spectrometers at external magnetic fields of 14.1 T (ν_0 = 600.1 MHz) and 23.4 T (ν_0 = 1 GHz), respectively. Measurements were carried out in 1.3 mm HFXY MAS triple-resonance probes with commercial ZrO_2 rotors at a rotation frequency of 62.5 kHz. RF nutation frequency was set to 200 kHz / 150 kHz, with pulse lengths of 1.1 µs / 1.7 µs, 128 scans / 64 scans, a recycle delay of 10 seconds for ν_0 = 600.1 MHz and a recycle delay optimized on the sample for measurements at ν_0 = 1 GHz. 2D homonuclear ^1H correlation spectra (ν_0 = 600.1 MHz) were recorded using the double-quantum (DQ) recoupling sequence $R12_2^5$ composed of 180° pulses with a recoupling time of 128µs.[11,12]

^{91}Zr NMR spectra were recorded at a magnetic field of 14.1 T (ν_0 = 55.79 MHz) at lowered temperature of approximately 120 K in a static triple resonance HFXY wide bore probe. Acquisition of spectra was performed by qCPMG sequence with 160’000 scans per spectrum and 32 echoes acquired per scan.[13–15] Recycle delay was set to 0.25 s. For accumulation of the whole spectrum, successive measurements with offsets of 50 kHz each were recorded (variable offset cumulative spectra). ^{91}Zr NMR spectroscopic experiments did not reveal any significant Zr background.

^{13}C and ^{15}N CPMAS NMR spectra were recorded on a Bruker AvanceIII HD spectrometer with an external magnetic field of 9.4 T and Larmor frequency of $\nu(^{13}C)$ = 100.58 MHz and $\nu(^{15}N)$ = 60.83 MHz, respectively. Measurements were carried out in a 3.2 mm HFXY MAS triple-resonance wide bore probe with spinning speed of 12.5 kHz (^{13}C) and 10 kHz (^{15}N). For the measurements initial ^1H nutation frequencies were set to 100 kHz, with a pulse length of 2.6 µs for initial $\pi/2$ pulse. The contact times and nutation frequencies of the ^{13}C channel were set to 3 ms and 62.5 kHz, while for ^{15}N CPMAS a contact time of 5 ms and approximately 60 kHz was utilized. For the ^1H channel the field strength was ramped linearly from 50 to 70 kHz.[16] Heteronuclear decoupling was performed with SPINAL-64 sequence[17] and a ^1H nutation frequency of 65 kHz with phase increments of 5 degrees and pulse lengths of 5.9 µs. A recycle delay of 2 s

was used with 32’768 scans for ^{13}C CPMAS and 153’664 scans for ^{15}N CPMAS, respectively. All ^{1}H and ^{13}C spectra are referenced indirectly with respect to tetramethylsilane (TMS) using adamantine as secondary reference and ^{15}N spectra were referenced to ammonium nitrate.

DFT-D plane-wave calculations

The DFT-D calculations were carried out using the CASTEP 17.2 code.[18] The GGA with PBE functional was used.[19] A plane-wave basis set was utilized with an energy cutoff of 600 eV, and the electrons were represented by ultrasoft pseudopotentials. k points were distributed using a Monkhorst-Pack-grid[20] with spacing of 0.03 $Å^{-1}$. Tkatchenko-Scheffler semiempirical dispersion correction was employed.[21] NMR parameters were calculated using the GIPAW approach.[22]

Quantum-Chemical Calculations on Molecular Model Systems

Depicted computational models were built from structures obtained by total scattering pair distribution function (PDF) analysis of *d*PCN-224. Structures for presented model compounds were optimized on PBE0-D3/def2-mSVP[23–26] level of theory. Subsequent frequency calculations were performed on the same level of theory to ensure all minima to be true minima on the potential energy hypersurface, where indicated. All calculations were carried out using Turbomole in version V7.3.[27]

1.3. Synthetic procedures

Synthesis $Zr_6O_4(OH)_4(Bz)_{12}$ cluster

The cluster was synthesized based on reported procedures with some changes.[28,29] 70 wt% $Zr(OPr)_4$ in 1-propanol (4.56 mL, 10.2 mmol) and benzoic acid (26.7 g, 0.218mmol) were added to 1-propanol (80 mL) in a round bottom flask (250 mL). The solution was sonicated for 20 min. The reaction mixture was heated in an oil bath (15 h, 110 °C) under reflux and stirring. The solvent was removed under vacuum at 40 °C. The precipitate was intensively washed with 1-propanol over a suction filter, dried at 40 °C, and identified as $Zr_6O_4(OH)_4(OBz)_{12}(PrOH)\cdot 4BzOH$ (short $Zr_6O_4(OH)_4Bz_{12}$) cluster via PXRD analysis.

Synthesis of MOF_$ZrCl_4$ (according to literature)

The synthesis was carried out according to a procedure published by Feng. et al.[30] with prolonged synthesis time for increased crystallinity. $ZrCl_4$ (7.0 mg, 0.03 mmol), $TCPPH_4$ (10.0 mg, 0.0127 mmol), and seven drops of acetic acid were ultrasonically dissolved in DEF (2.0 mL) in an 8 dram glass vial. The mixture was heated in an oven (24 h, 120 °C). The reaction product was recovered by centrifugation and left to dry at 60 °C overnight.

Half of the product (4.0 mg) was resuspended in DMF (2.0 mL) and activated with HCl (8M, 50 µL) in an oven (15 h, 100 °C). The product was collected by centrifugation and washed with DMF (three times, 16k rpm/20 min/16 °C) and acetone (twice, 16k rpm/8 min/16 °C). The mixture was soaked in acetone overnight and washed with acetone one more time. Centrifugation (16k rpm/5 min/16 °C) and drying for 3 h at room temperature yielded a purple powder as product.

Synthesis of MOF_ZrOCl$_2$

ZrOCl$_2$·8H$_2$O (98.8 mg, 0.307 mmol) and benzoic acid (1.30 g, 10.6 mmol) were solved in DMF (18 mL) in a 20 mL microwave vial by sonication for 2 min and heated in a microwave (1 h, 100 °C) under stirring. After cooling, TCPPH$_4$ (44.8 mg, 0.0567 mmol) was added and dissolved *via* sonication. The reaction mixture was heated in a microwave (1 h, 130 °C) under stirring, washed with DMF (three times, 16k rpm/15 min/16 °C) and resuspended in DMF (10 mL). The suspension was activated with HCl (8M, 0.20 mL) in an oven (15 h, 100 °C). The product was collected by centrifugation and washed with DMF (three times, 16k rpm/20 min/16 °C) and acetone (twice, 16k rpm/8 min/16 °C). The mixture was soaked in acetone overnight and washed with acetone one more time. Centrifugation (16k rpm/5 min/16 °C) and drying for 3 h at room temperature yielded a purple powder as product.

Synthesis of MOF_ZrOCl$_2$_(II)

For comparison, an additional PCN-221 (*d*PCN-224) sample was synthesized from ZrOCl$_2$·8H$_2$O derived from a PCN-224 protocol[32]. ZrOCl$_2$·8H$_2$O (120 mg, 0.372 mmol), TCPPH$_4$ (40.0 mg, 0.0506 mmol) and benzoic acid (1.30 g, 10.6 mmol) were dissolved in DMF (8.0 mL) in a 20 mL microwave vial *via* sonication. The solution was heated in an oven (24 h, 120 °C), washed with DMF (three times, 16k rpm/15 min/ 16 °C) and resuspended in DMF (10 mL). The reaction mixture was activated with HCl (8M, 0.50 mL) in an oven (15 h, 100 °C). The suspension was washed with DMF (twice, 16k rpm/20 min/16 °C) and acetone (twice, 16k rpm/8 min/ 16 °C). The mixture was soaked in acetone overnight and washed with acetone one more time. The product was obtained as purple powder.

Synthesis of MOF_Zr$_6$

Zr$_6$O$_4$(OH)$_4$Bz$_{12}$ cluster (138 mg, 0.372 mmol), TCPPH$_4$ (44.8 mg, 0.0567 mmol) and benzoic acid (1.20 g, 9.83 mmol) were dissolved in DMF (8.0 mL) in a 20 mL microwave vial *via* sonication. The solution was heated in an oven (24 h, 120 °C), washed with DMF (three times, 16k rpm/15 min/16 °C) and resuspended in DMF (10 mL). The reaction mixture was activated with HCl (8M, 1.0 mL) in an oven (15 h, 100 °C). The suspension was washed with DMF (twice, 16k rpm/20 min/16 °C) and acetone (twice,

16k rpm/8 min/16 °C). The mixture was soaked in acetone overnight and washed with acetone one more time. The product was obtained as purple powder.

Synthesis of experimental PCN-224

The synthesis was carried out based on a MOF-525 procedure published by Morris et al.[31] in which PCN-224 was yielded instead of MOF-525. $ZrOCl_2 \cdot 8H_2O$ (188 mg, 0.582 mmol) and $TCPPH_4$ (37.5 mg, 0.047 mmol) were dissolved in DMF (150 mL) in a 500 mL laboratory bottle *via* sonication. Acetic acid (37.0 mL, 647 mmol) was added and the mixture was sonicated again. The reaction mixture was heated in an oven (72 h, 65 °C), washed with DMF (twice, 16k rpm/15 min/16 °C) and resuspended in DMF (10 mL). The suspension was activated with HCl (8M, 0.50 mL) in an oven (15 h, 100°C). The product was collected by centrifugation and washed with DMF (twice, 16k rpm/20 min/16 °C) and acetone (twice, 16k rpm/8 min/16 °C). The mixture was soaked in acetone overnight and washed with acetone one more time. Centrifugation (16k rpm/5 min/16 °C) and drying for 3 h at room temperature yielded a purple powder as product.

Synthesis of Single Crystal

$ZrOCl_2 \cdot 8H_2O$ (30 mg, 0.093 mmol), $TCPPH_4$ (10.0 mg, 0.013 mmol) and benzoic acid (700 mg, 5.71 mmol) were dissolved in DMF (2.0 mL) in an 8 dram glass vial *via* sonication. The solution was heated in an oven (24 h, 120 °C), washed with DMF (three times, 16k rpm/15 min/16 °C) and resuspended in DMF (10 mL). The reaction mixture was activated with HCl (8M, 0.50 mL) in an oven (15 h, 100°C). The suspension was washed with DMF (twice, 16k rpm/20 min/16 °C) and acetone (twice, 16k rpm/8 min/16 °C). The mixture was soaked in acetone overnight washed with acetone one more time. Dark purple powder was obtained as product, composed of 10 – 20 µm big cubic crystals and needle-shaped crystals identified as PCN-222 impurities.

2. Results and discussion

2.1. SEM Images

Scanning electron microscopy (SEM) images of all samples show distinctively different crystal shapes and sizes ranging from cuboctahedra/truncated cubes (MOF_$ZrCl_4$ and MOF_$ZrOCl_2$) to perfectly shaped cubes (MOF_$ZrOCl_2$_(II)), whereas no particular morphology can be identified for the crystallites of MOF_Zr_6 due to the very small size of the particles (Figure S1). We attribute the apparent differences of the particles sizes and morphologies to the different modulators (acetic acid vs. benzoic acid) and relative concentrations in the synthesis, which regulate both the nucleation and growth of MOF particles and not to different crystal structures.[33–35] For comparison, PCN-224 was imaged as well, which consists of cubic particles (Figure S2).

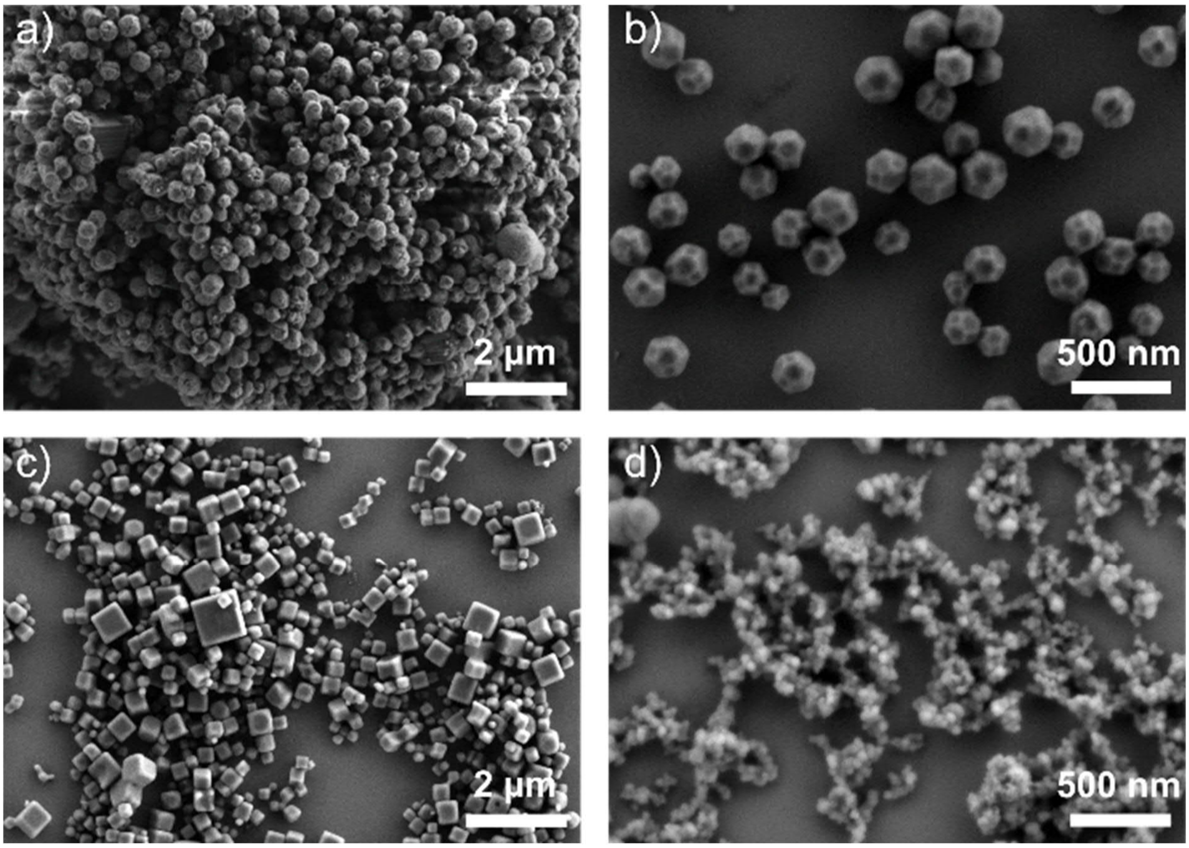

Figure S1: Scanning electron microscopy (SEM). SEM images of a) MOF_$ZrCl_4$, b) MOF_$ZrOCl_2$, c) MOF_$ZrOCl_2$_(II), and d) MOF_Zr_6.

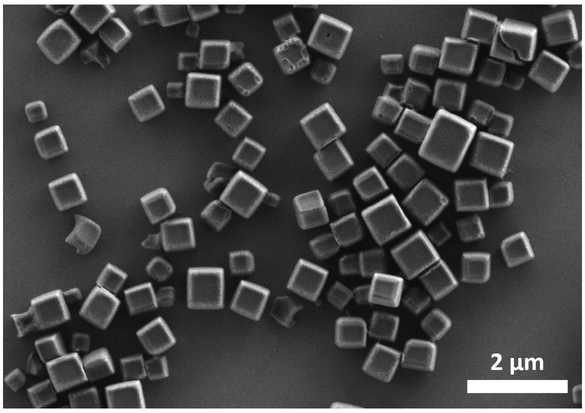

Figure S2: Scanning electron microscopy image of experimental PCN-224.

2.2. Powder X-Ray Diffraction

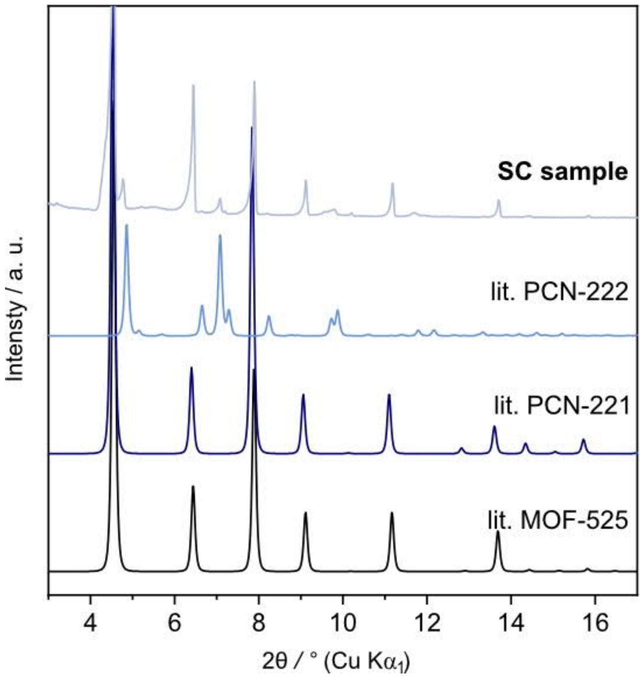

Figure S3: Powder X-ray diffraction (PXRD) pattern of single crystal. PXRD pattern of the sample containing the measured single crystal compared to the simulated patterns (lit.) of MOF-525, PCN-221, and PCN-222.[30,31,36]

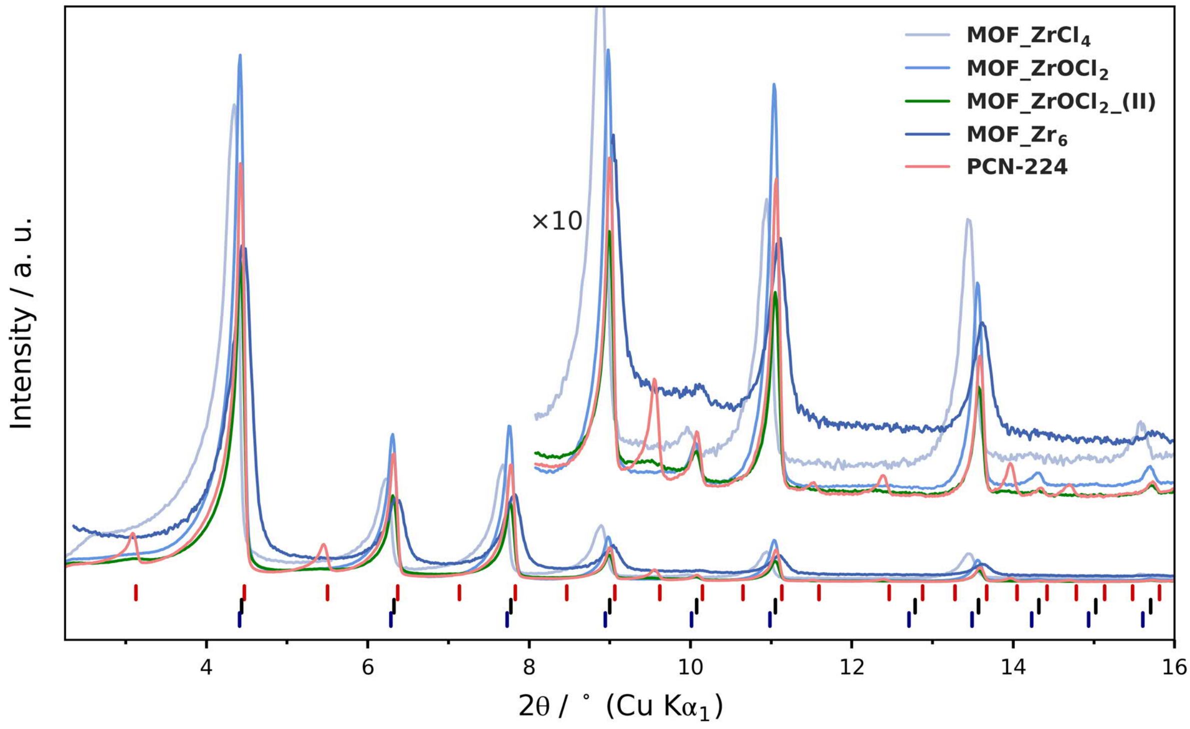

Figure S4: Comparison of powder X-ray diffraction patterns for all samples. The patterns are also multiplied by a factor of ten beyond 8° 2θ to show a zoomed comparison of the patterns. The reflection positions for published structures are given below for reference: PCN-224 (red), MOF-525 (black), PCN-221 (purple).

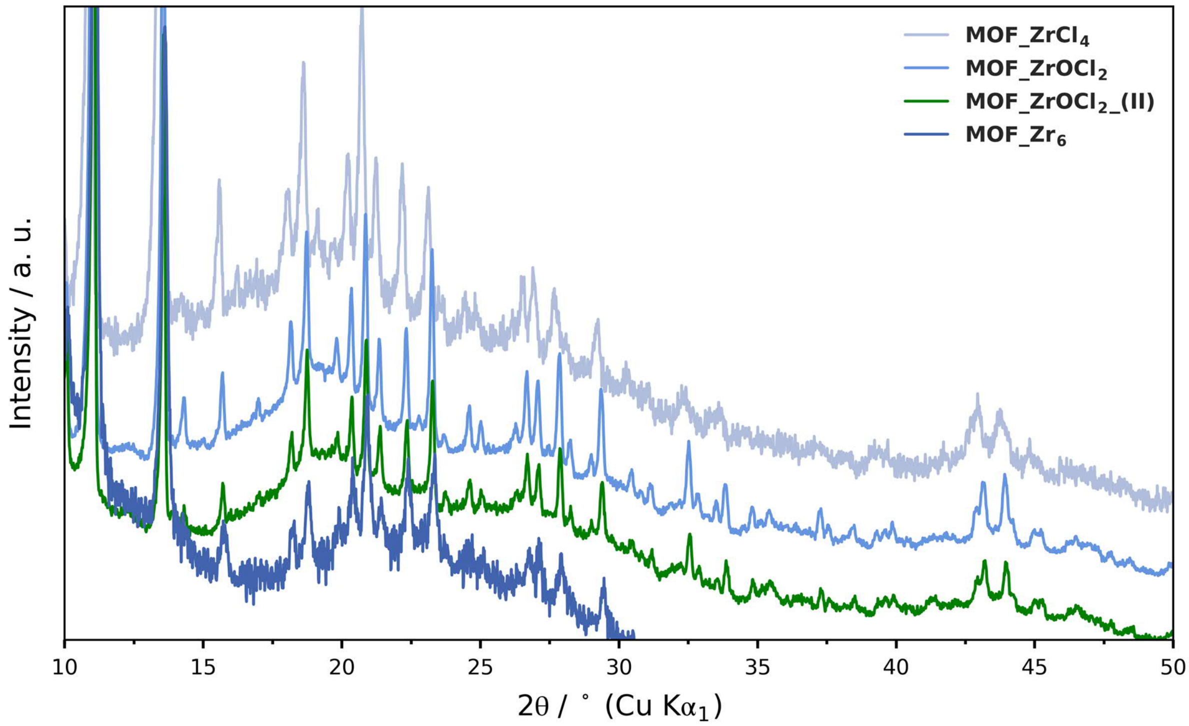

Figure S5: Comparison of powder X-ray diffraction patterns at high ranges. Comparison for MOF_ZrCl$_4$, MOF_ZrOCl$_2$, MOF_ZrOCl$_2$_(II), and MOF_Zr$_6$ zoomed into a high 2θ range to show the similarity in the high-angle diffraction features. Peak positions and relative intensities match well, although there are some differences for example a small peak in Lit at approximately 19°. MOF_Zr$_6$ was only measured up to 30°.

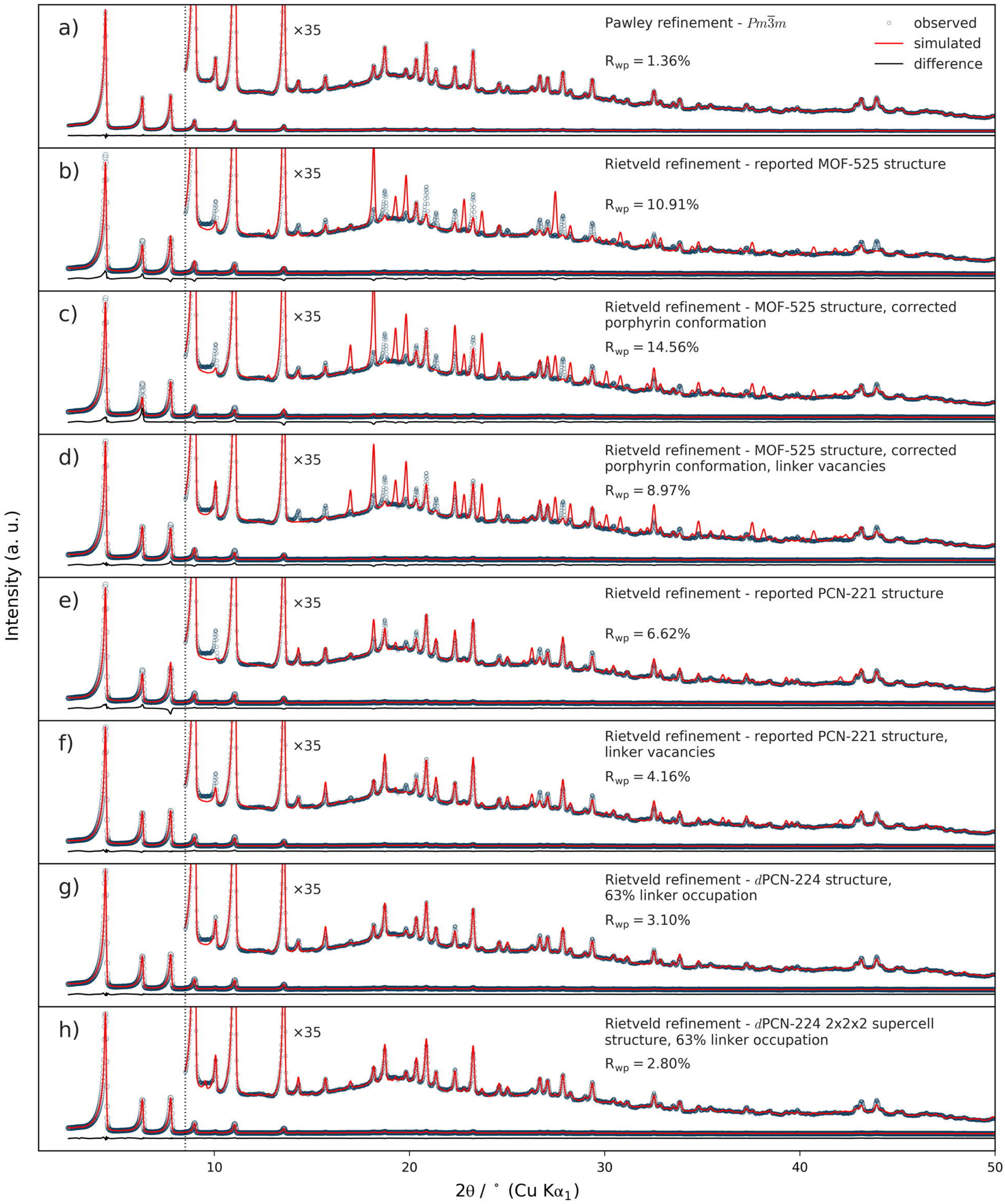

Figure S6: Pawley and Rietveld refinements of sample MOF_$ZrOCl_2$. a) Resulting fit from Pawley refinement to the diffraction pattern of MOF_$ZrOCl_2$ using the published cell with $Pm\bar{3}m$ symmetry. Rietveld refinements to the same sample data are shown for b) published MOF-525 structure, c) MOF-525 with twisted linker conformation, d) MOF-525 with twisted linker conformation and linker vacancies, e) published PCN-221 structure with Zr_8 clusters, f) published PCN-221 structure with Zr_8 clusters and linker vacancies, g) *d*PCN-224-1 structure with 25% occupancy of each cluster orientation, twisted linker conformations, and linker vacancies, and h) 2x2x2 *d*PCN-224-2 supercell model, which allows refinement of separate occupancies for each individual linker and cluster orientation for each site. All models considered the occupancy of pseudo-atom densities at additional open sites on the cluster, which were accounted for primarily by additional water and possibly other leftover coordinating species. For this sample, both *d*PCN-224 models lead to linker occupation of 63%, i.e. ~7.5 linkers per cluster.

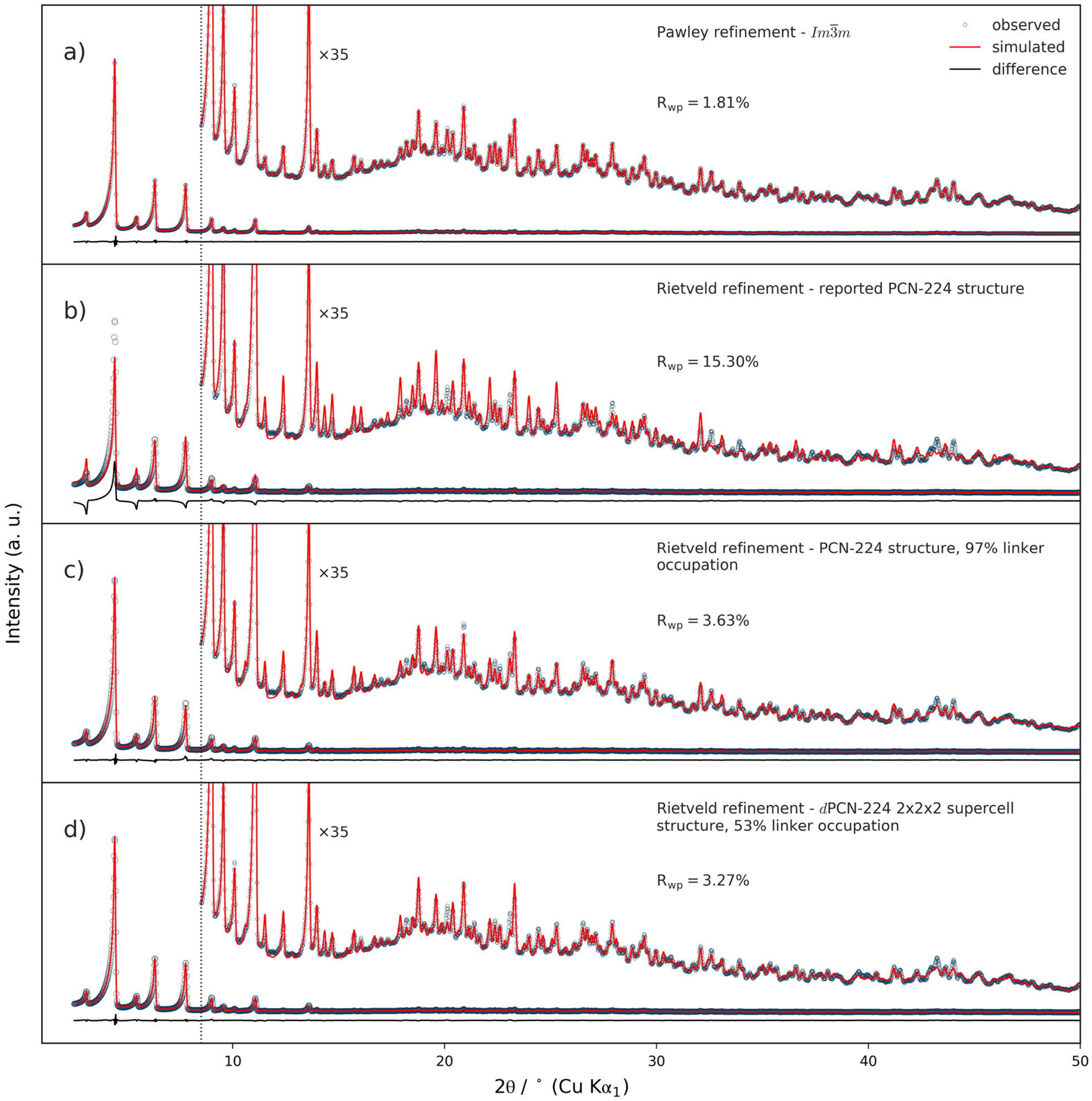

Figure S7: Pawley and Rietveld refinements of sample PCN-224. a) Resulting fit from Pawley refinement to the diffraction pattern of PCN-224 using the published cell with *Im*–3*m* symmetry. Rietveld refinements to the same sample data are shown for b) published PCN-224 structure, c) PCN-224 with additional linker vacancies and modulator densities, and d) the *d*PCN-224-2 supercell model from Figure S 6(h). The PCN-224 model gave 97% linker occupation of 6 possible sites, i.e. ~5.8 linkers per cluster. In this case, the extra pseudo-atoms refined to the typical vacant tetrakis(4-carboxyphenyl) porphyrin (TCPP) sites of PCN-224. The supercell model gave slightly different occupations, i.e. ~6.4 linkers per cluster, with pseudo-atom sites on the 6 additional sites between TCPP positions.

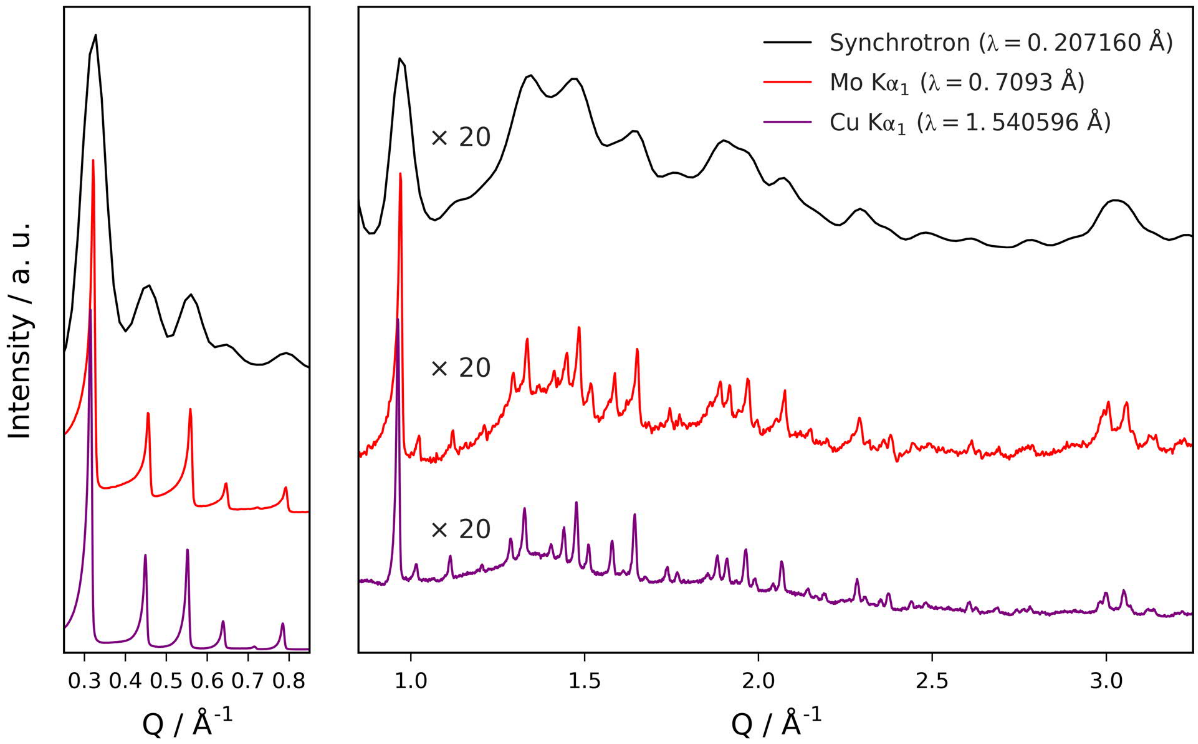

Figure S8: Powder X-ray powder diffraction (PXRD) of sample MOF_$ZrOCl_2$_(II). PXRD intensities measured from the same MOF_$ZrOCl_2$_(II) sample (1 mm ID polyimide capillary) using synchrotron, Mo $K\alpha_1$, and Cu $K\alpha_1$ radiation. Broadening of the Bragg peaks occurs due to the increase in incident photon energies, and in the case of the synchrotron measurements, due also to decreased resolution of the detector.

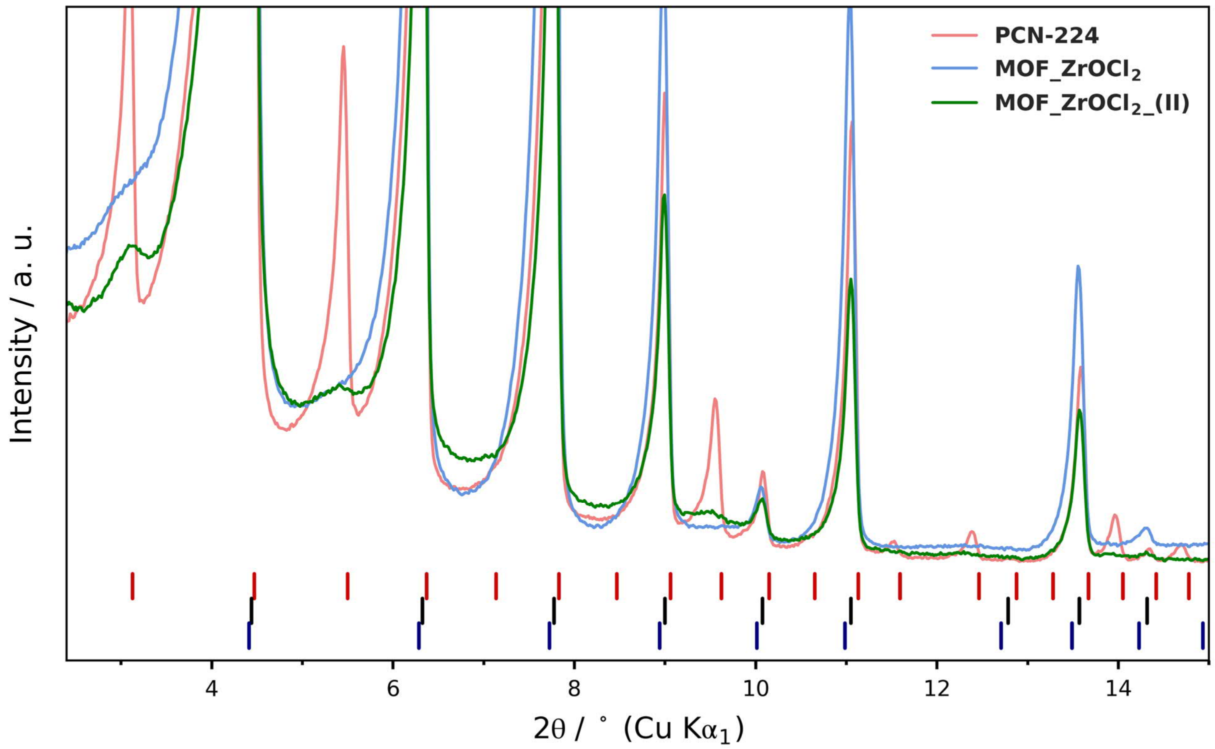

Figure S9: Powder X-ray diffraction (PXRD) of all samples at low angles. Since the pair distribution function (PDF) refinements suggest occupational disorder of the linkers at the level of about 50–60% occupancy, we show again the PXRD patterns measured for the four samples, but zoomed closely into the low angle region. PCN-224 has two extra distinct peaks at approximately 3.1 and 5.4° 2θ, which help distinguish the *Im*–3*m* symmetry from *Pm*–3*m*. However, upon close inspection we can see that there is also very small peaks at these angles for the MOF_ZrOCl$_2$_(II) sample, and further, e.g. 9.6°. The MOF_ZrOCl$_2$ sample also shows additional diffuse intensities. This suggests that linker vacancies are random, rather than ordered in *Im*–3*m* symmetry as with PCN-224, therefore only producing diffraction effects with only limited spatial coherence.

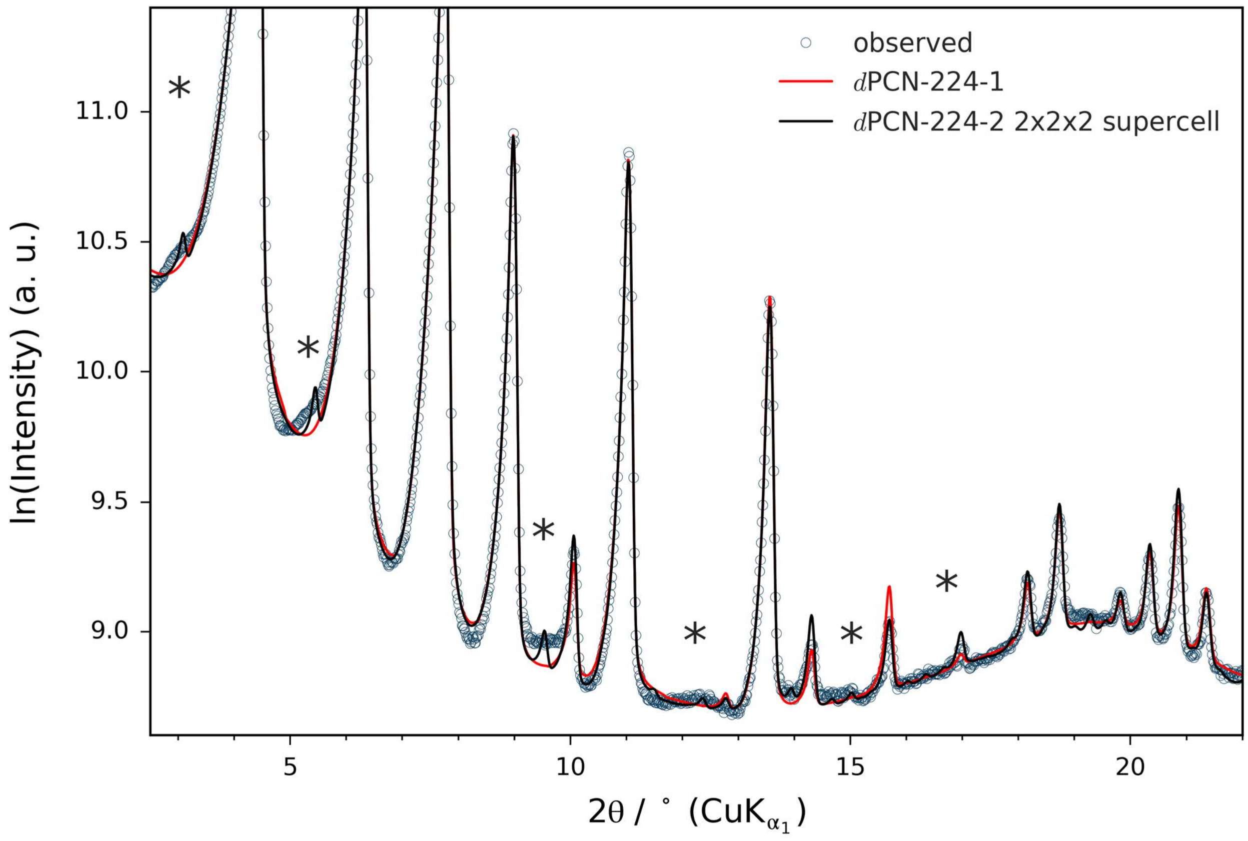

Figure S10: Overlay of the Rietveld refinements of *d*PCN-224 models to the observed MOF_ZrOCl$_2$ pattern. The refinements here are from the single cell *d*PCN-224-1 model (red) and the 2x2x2 supercell *d*PCN-224-2 model (black). The fit for the supercell was performed using a *P*1 symmetry, and separate occupancy factors have been set for each individual porphyrin linker and each individual cluster orientation for all eight cluster sites. We see that the supercell, with lattice parameter similar to PCN 224, allows for the modeling of the additional weak intensities in the peaks at approx. 2.8, 5.1, 9.4, 12° 2θ and further, marked by black asterisks (*). In the experimental data, the peaks are broader indicating the limited spatial coherence of these features. Overall, the supercell is still only a limited expansion of the possibilities for types of linker vacancies/cluster orientation patterns which may occur locally. However, this supports the plausibility for this frustrated network picture of the structure to explain the observed data.

2.3. Chemical Composition Analysis

Chemical analysis was performed for MOF_$ZrCl_4$, MOF_$ZrOCl_2$, MOF_$ZrOCl_2$_(II), MOF_Zr_6, and experimental PCN-224. The theoretical and experimental weight percent of Zr, C, H, and N and the calculated Zr/C and Zr/N ratios are given in Table S1–S5. For comparison, the percentage of vacancies was calculated as function of the Zr/C or Zr/N ratio considering either modulator (benzoic acid (BzA) or acetic acid (AcA)) on the TCPP vacant sites or hydroxy groups with additional water in the pores. The polynomial fits of the Zr/C and Zr/N ratios with the amount of TCPP vacancies x, are shown in Figure S11–S13. We found high amounts of TCPP vacancies for all samples, including experimental PCN-224 which is reported with only 50% TCPP linkers.[37] However, solvent molecules such as water, DMF, and Acetone may remain in the pores of the MOFs, which may falsify the calculated percentage of defects.

Table S1: Calculated chemical composition and amount of linker vacancies of $Zr_6O_4(OH)_4(TCPP)_{3-x}(BzA)_x$, where x represents the amount of tetrakis(4-carboxyphenyl) porphyrin vacancies replaced by benzoic acid.

x	3-x	Vacancies [%]	Zr [wt%]	C [wt%]	H [wt%]	N [wt%]	Zr/C	Zr/N
0.00	3.00	0.00	17.3	57.4	2.77	5.32	0.30	3.26
0.60	2.40	20.0	20.7	54.3	2.65	5.09	0.38	4.07
0.70	2.30	23.3	21.3	53.8	2.64	5.01	0.40	4.25
0.80	2.20	26.7	21.8	53.3	2.62	4.92	0.41	4.44
0.90	2.10	30.0	22.4	52.7	2.61	4.82	0.43	4.65
1.00	2.00	33.3	23.1	52.1	2.59	4.72	0.44	4.89
1.10	1.90	36.7	23.7	51.5	2.57	4.61	0.46	5.15
1.20	1.80	40.0	24.4	50.8	2.56	4.50	0.48	5.43
1.30	1.70	43.3	25.2	50.1	2.54	4.38	0.50	5.75
1.40	1.60	46.7	26.0	49.4	2.52	4.25	0.53	6.11
1.50	1.50	50.0	26.8	48.6	2.49	4.12	0.55	6.51

Table S2: Calculated chemical composition and amount of linker vacancies of $Zr_6O_4(OH)_4(TCPP)_{3-x}(AcA)_x$, where x represents the amount of tetrakis(4-carboxyphenyl) porphyrin vacancies replaced by acetic acid.

x	3-x	Vacancies [%]	Zr [wt%]	C wt%]	H [wt%]	N [wt%]	Zr/C	Zr/N
0.00	3.00	0.00	17.7	56.6	2.77	5.42	0.31	3.26
0.60	2.40	20.0	21.0	53.7	2.64	5.17	0.39	4.07
0.70	2.30	23.3	21.6	53.1	2.63	5.09	0.41	4.25
0.80	2.20	26.7	22.3	52.4	2.61	5.02	0.43	4.44
0.90	2.10	30.0	23.0	51.7	2.59	4.93	0.44	4.66
1.00	2.00	33.3	23.7	50.9	2.57	4.85	0.46	4.88
1.10	1.90	36.7	24.4	50.1	2.55	4.75	0.49	5.15
1.20	1.80	40.0	25.3	49.2	2.53	4.66	0.51	5.42
1.30	1.70	43.3	26.1	48.3	2.51	4.55	0.54	5.75
1.40	1.60	46.7	27.1	47.3	2.48	4.44	0.57	6.10
1.50	1.50	50.0	28.1	46.2	2.46	4.31	0.61	6.52

Table S3: Calculated chemical composition and amount of linker vacancies of $Zr_6O_4(OH)_4(TCPP)_{3-x}(OH)_x(H_2O)_{12}$, where x represents the amount of tetrakis(4-carboxyphenyl) porphyrin vacancies replaced by hydroxyl groups.

x	3-x	Vacancies [%]	Zr [wt%]	C wt%]	H [wt%]	N [wt%]	Zr/C	Zr/N
0.00	3.00	0.00	16.72	52.85	3.30	5.14	0.316	3.25
0.10	0.90	3.33	17.22	52.60	3.28	5.11	0.327	3.37
0.20	0.80	6.67	17.65	52.04	3.28	5.06	0.339	3.49
0.30	2.70	10.0	18.09	51.46	3.28	5.00	0.352	3.62
0.40	2.60	13.3	18.57	50.85	3.28	4.94	0.365	3.76
0.50	2.50	16.7	19.06	50.20	3.28	4.88	0.380	3.91
0.60	2.40	20.0	19.59	49.52	3.28	4.81	0.396	4.07
0.70	2.30	23.3	20.15	48.80	3.28	4.74	0.413	4.25
0.80	2.20	26.7	20.73	48.04	3.28	4.67	0.432	4.44
0.90	2.10	30.0	21.36	47.24	3.28	4.59	0.452	4.65
1.00	2.00	33.3	22.02	46.38	3.28	4.51	0.475	4.88
1.10	1.90	36.7	22.72	45.47	3.28	4.42	0.500	5.14
1.20	1.80	40.0	23.47	44.50	3.28	4.32	0.527	5.43
1.30	1.70	43.3	24.27	43.46	3.29	4.22	0.558	5.75
1.40	1.60	46.7	25.13	42.35	3.29	4.12	0.593	6.10
1.50	1.50	50.0	26.05	41.16	3.29	4.00	0.633	6.51

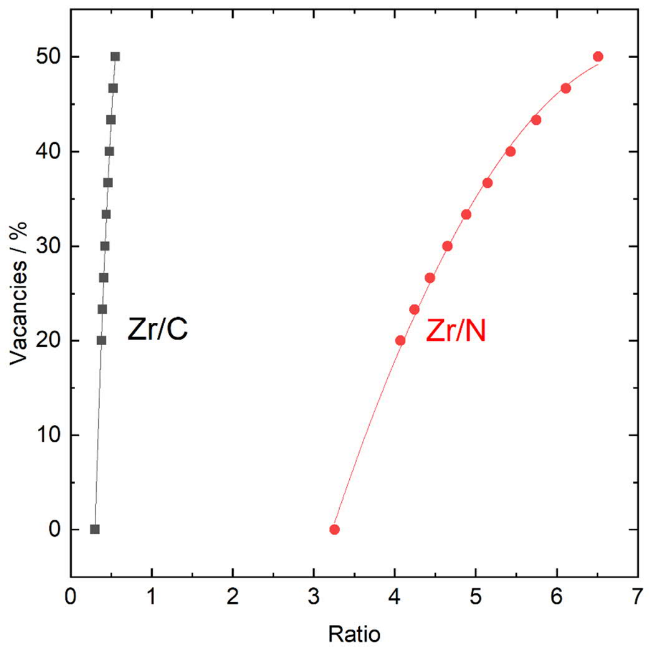

Figure S11: Percentage of vacancies replaced by the modulator benzoic acid (BzA). Vacancies as a function of Zr/C ratio (black) and Zr/N ratio (red) in $Zr_6O_4(OH)_4(TCPP)_{3-x}(BzA)_x$ were x represents the amount of tetrakis(4-carboxyphenyl) porphyrin (TCPP) vacancies, replaced by modulator: x = 0 (0 % vacancies) and 0.6 (20% vacancies) < x < 1.5 (50% vacancies). Polynomial fit for Zr/C (black): $y = -116.08 + 484.69x + (-333.07)x^2$ and for Zr/N (red): $y = -115.61 + 46.165x + (-3.2027)x^2$.

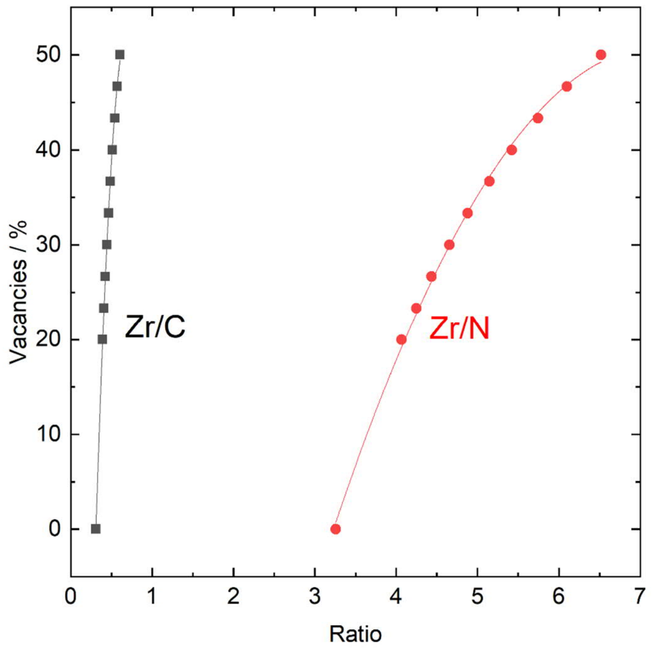

Figure S12: Percentage of vacancies replaced by the modulator acetic acid (AcA). Vacancies as a function of Zr/C ratio (black) and Zr/N ratio (red) in $Zr_6O_4(OH)_4(TCPP)_{3-x}(AcA)_x$ were x represents the amount of tetrakis(4-carboxyphenyl) porphyrin (TCPP) vacancies, replaced by modulator: x = 0 (0% vacancies) and 0.6 (20% vacancies) < x < 1.5 (50% vacancies). Polynomial fit for Zr/C (black): $y = -117.7 + 487.4x + (-349.5)x^2$ and Zr/N (red): $y = -116.2 + 46.39x + (-3.223)x^2$.

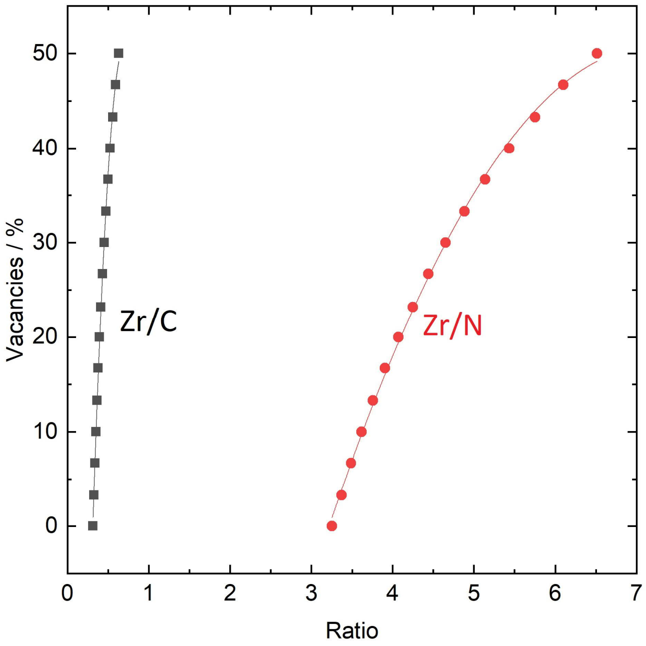

Figure S13: Percentage of vacancies replaced by hydroxyl groups. Vacancies as a function of Zr/C ratio (black) and Zr/N ratio (red) in $Zr_6O_4(OH)_4(TCPP)_{3-x}(OH)_x(H_2O)_{12}$ were x represents the amount of tetrakis(4-carboxyphenyl) porphyrin (TCPP) vacancies, replaced by hydroxy groups: x = 0 (0% vacancies) < x < 1.5 (50% vacancies). Polynomial fit for Zr/C (black): $y = -115.1 + 474.2x + (-339.1)x^2$ and Zr/N (red): $y = -114.9 + 45.99x + (-3.193)x^2$.

Table S4: Particle sizes determined from scanning electron microscopy images and chemical compositions of synthesized MOFs.

	Size [nm]	Zr [wt%]	C [wt%]	H [wt%]	N [wt%]	Zr/C	Zr/N
MOF_$ZrCl_4$	427(111)	19.82	48.25	3.84	4.63	0.411	4.28
MOF_$ZrOCl_2$	182(34)	21.1	46.27	3.49	4.01	0.456	5.26
MOF_$ZrOCl_2$_(II)	233(117)	22.3	42.50	3.45	3.92	0.525	5.69
MOF_Zr_6	39(11)	23.3	46.90	3.31	4.50	0.496	5.17
Exp. PCN-224	469(129)	23.47	41.22	3.49	3.67	0.569	6.40

Table S5: Zr/C and Zr/N ratios obtained from chemical composition analysis of synthesized MOFs and calculated amount of linker vacancies with and without modulator on the vacant sites.

	Zr/C	Zr/N	Vacancies with mod. Zr/C [%]	Vacancies with mod. Zr/N [%]	Vacancies without mod. Zr/C [%]	Vacancies without mod. Zr/N [%]
MOF_$ZrCl_4$	0.411	4.28	23.6	23.3	22.5	23.4
MOF_$ZrOCl_2$	0.456	5.26	35.7	38.7	30.6	38.7
MOF_$ZrOCl_2$_(II)	0.525	5.69	46.6	43.4	40.4	43.4
MOF_Zr_6	0.496	5.17	42.4	37.5	36.7	37.5
Exp. PCN-224	0.569	6.40	46.5	48.7	44.9	48.7

2.4. Pair distribution function analysis

The Zr–Zr pair distance reported for the Zr_8O_6 cluster in the PCN-221, structure reported by Feng et al.[30], is r = 2.688 Å. This is significantly contracted when compared to the Zr–Zr distance expected, for instance, for the Zr_6 cluster. To get a sense for the likelihood of the Zr_8 Zr–Zr distance to exist, we performed a meta-analysis of the Zr–Zr nearest neighbor (NN) pair distances reported for both other metal–organic structures obtained from the Cambridge Crystallographic Data Centre (CCDC) and inorganic structures obtained from the Inorganic Crystal Structure Database (ICSD). CCDC structures were obtained from the WebCSD portal. A formula search was performed with the following criteria (Zr > 1, O > 1, C > 1, H > 1), netting at the time 472 structures. Further filtering of structures was determined by the following criteria. It was determined that all structures containing Zr–O bond distances below 1.8 contained spurious structural errors or disordered sites which do not make sense in a true local structural sense, and were not considered *(CCDC 1858054, CCDC 1006913, CCDC 1976030, CCDC 1588089, CCDC 1858052, CCDC 1838332, CCDC 1838334, CCDC 955328, CCDC 1580358, CCDC 1587886, CCDC 1908939, CCDC 1908940).* Structures which contained Zr–Zr distances below 3.0 were determined to contain disordered sites or Zr_8-type clusters, and were not included *(CCDC 1955286, CCDC 1850690, CCDC 1486878, CCDC 1471480, CCDC 1487038).* Structures with Zr–Zr distances above 4.0, which were not connected by an oxygen, were not included, and some structures contained no atomic coordinates. Structures from the ICSD were filtered by structures that contain only Zr and O, and which were obtained at ambient pressure, and at 300 K or below. This resulted in 92 structures.

Bond distances for all structures were determined using the python code from xPDFsuite software program.[10] In the case of the CCDC structures, there are still some significant outliers, though we did not further check the remaining structures for quality markers, and other structures with mistakes or disordered sites may still be represented in the list. Either way, it is clear that the Zr–Zr distances for the Zr_8O_6 cluster is an extreme outlier, despite the representation of a wide variety of ZrO based molecular cluster stoichiometries. The case is the same for comparison to inorganic ZrO crystal structures, indicating that this Zr_8O_6 motif is highly unlikely based on known structuring behavior.

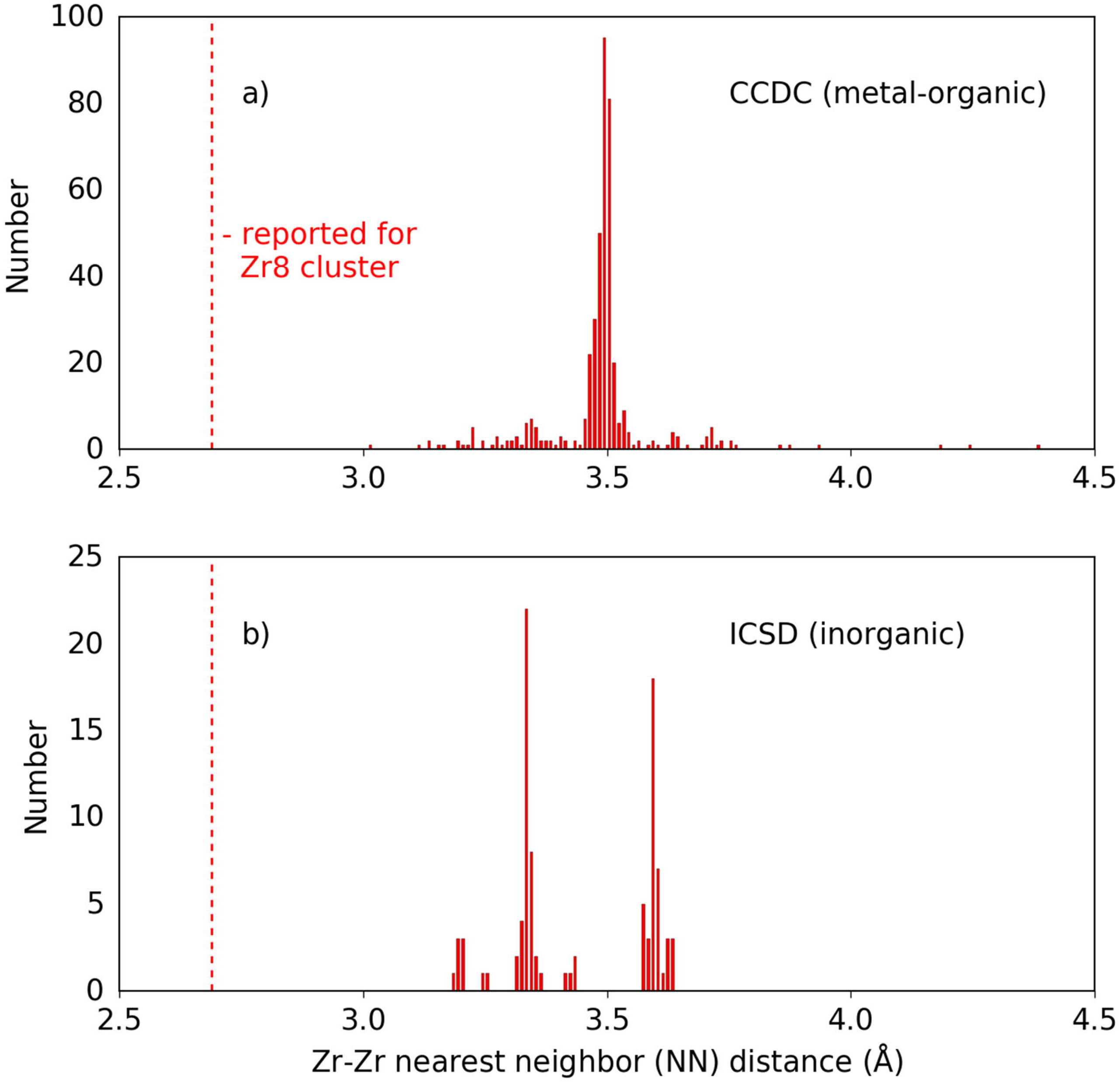

Figure S14: Distribution of first nearest neighbor (NN) Zr–Zr pair distances. Distribution observed in structures obtained from a) CCDC and b) ICSD structure databases. A red dashed line is shown indicating the Zr–Zr pair distance reported for the Zr_8 cluster, which is an extreme outlier.

Analysis of the PDF provides a useful method for interpreting structure information from total scattering data. It provides a sensitive probe of the local structure in amorphous and nanostructured materials, because it does not require symmetry, and treats both Bragg and diffuse scattering equally.[38] A diffraction measurement over a wide range of momentum transfer and with good statistics is required to obtain suitable PDFs for structure analysis. To obtain the PDF, the measured coherent powder diffraction intensities are first normalized by the average form factor squared to obtain the total scattering structure function $S(Q)$, defined as

$$S(Q) = \frac{I(Q) - \langle f(Q)^2 \rangle + \langle f(Q) \rangle^2}{\langle f(Q) \rangle^2}.$$

The experimental PDF, denoted $G(r)$, is the truncated Fourier transform of the reduced, total scattering structure function, $F(Q) = Q[S(Q) - 1]$, as

$$G(r) = \frac{2}{\pi} \int_{Q_{min}}^{Q_{max}} F(Q) sin(Qr) dQ,$$

where $G(r)$ is the magnitude of the scattering momentum transfer. For elastic scattering,

$$Q = 4\pi sin\theta/\lambda,$$

where λ is the probe wavelength and 2θ is the scattering angle. In practice, values of Q_{min} and Q_{max} are determined by the experimental setup, and Q_{max} is often reduced below the experimental maximum to reduce the effects of low signal-to-noise in the high-Q region on the Fourier transformation. To aid in qualitative assessment of the long-distance structural correlations, a modification function can be applied to $F(Q)$ prior to Fourier transformation by,

$$G(r) = \frac{2}{\pi} \int_{Q_{min}}^{Q_{max}} M(Q) F(Q) sin(Qr) dQ$$

and,

$$M(Q) = \frac{sin(Qr_{ij})}{Qr_{ij}}.$$

$M(Q)$ is called a modification function, in this case a Lorch function[39,40], which damps the intensity of $F(Q)$ to 0 at Q_{max}. This reduces the effects of termination from high-Q signal and noise intensities in the reduced structure function, which suppresses non-structural high frequency oscillations in the PDF. In this study, a Lorch function was only used for processing PDFs from the Mo $K\alpha_1$ measurements, for qualitative analysis.

The PDF gives the scaled probability of finding two atoms in a material a distance r apart and is relative to the density of atom pairs in the material. For a macroscopic scatterer, $G(r)$ is calculated from a known structure model according to

$$G(r) = 4\pi r[\rho(r) - \rho_0],$$

$$\rho(r) = \frac{1}{4\pi r^2 N}\sum_i \sum_{j\neq i} \frac{f_i f_j}{\langle f\rangle^2}\delta(r - r_{ij}).$$

Here, ρ_0 is the average number density of the material and ρ(r) is the local atomic pair density, which is the mean weighted density of neighbor atoms at distance r from an atom at the origin. The sums in $\rho(r)$ run over all atoms in the sample, f_i is the scattering factor of atom i, $\langle f\rangle$ is the average scattering factor and r_{ij} is the distance between atoms i and j. In this study, these equations were used to fit the PDF generated from a structure model to the experimental PDFs in using the program PDFgui.[41] The delta functions were Gaussian-broadened to account for atom displacements and the finite Q_{range} of the experiment, and the equation was modified to account for signal damping and broadening due to experimental effects. PDF modeling was performed by adjusting the lattice parameter *a,* atomic displacement parameters (ADPs) for Zr, O, and the linkers, correlated motion of neighboring atoms using *rcut* and *sratio* parameters to sharpen peaks within cluster or linker components, domain size (*spdiameter*), and a global scale factor. The refinements were run by minimizing R_w, calculated as

$$R_w = \sqrt{\frac{\sum_{i=1}^{n}[G_{obs}(r_i) - G_{calc}(r_i, P)]^2}{\sum_{i=1}^{n} G_{obs}(r_i)^2}},$$

which was used to quantify the goodness-of-fit for the model.

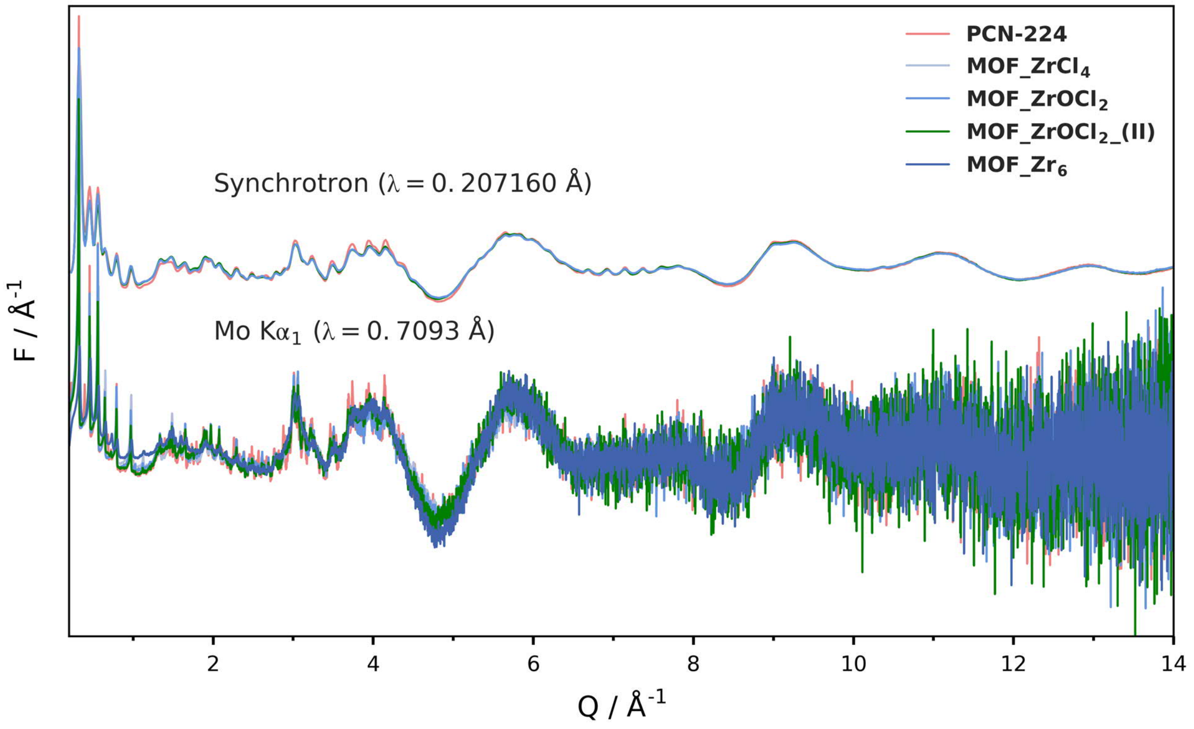

Figure S15: Reduced X-ray total scattering $\boldsymbol{F(Q)}$. Patterns are shown as obtained from both synchrotron and Mo Kα_1 measurements. The signal-to-noise is significantly improved in the synchrotron data.

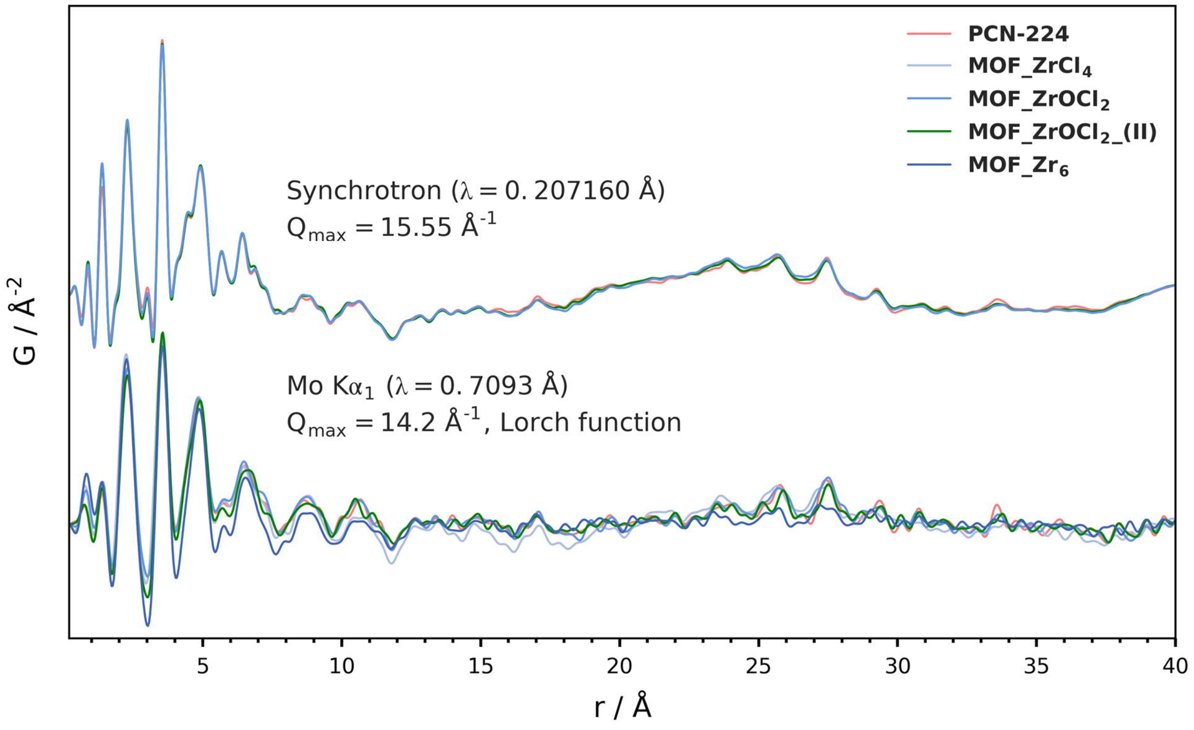

Figure S16: Pair distribution functions $\boldsymbol{G(r)}$ (PDFs) of all samples. PDFs shown as obtained from both synchrotron and Mo Kα_1 measurements. The Mo Kα_1 PDFs were obtained by Fourier transformation of the reduced structure function after multiplication with a Lorch function to reduce the effects of low signal-to-noise at high Q. There is good agreement between the PDFs obtained for all samples, indicating that the local and intermediate range ordering is very similar for all samples.

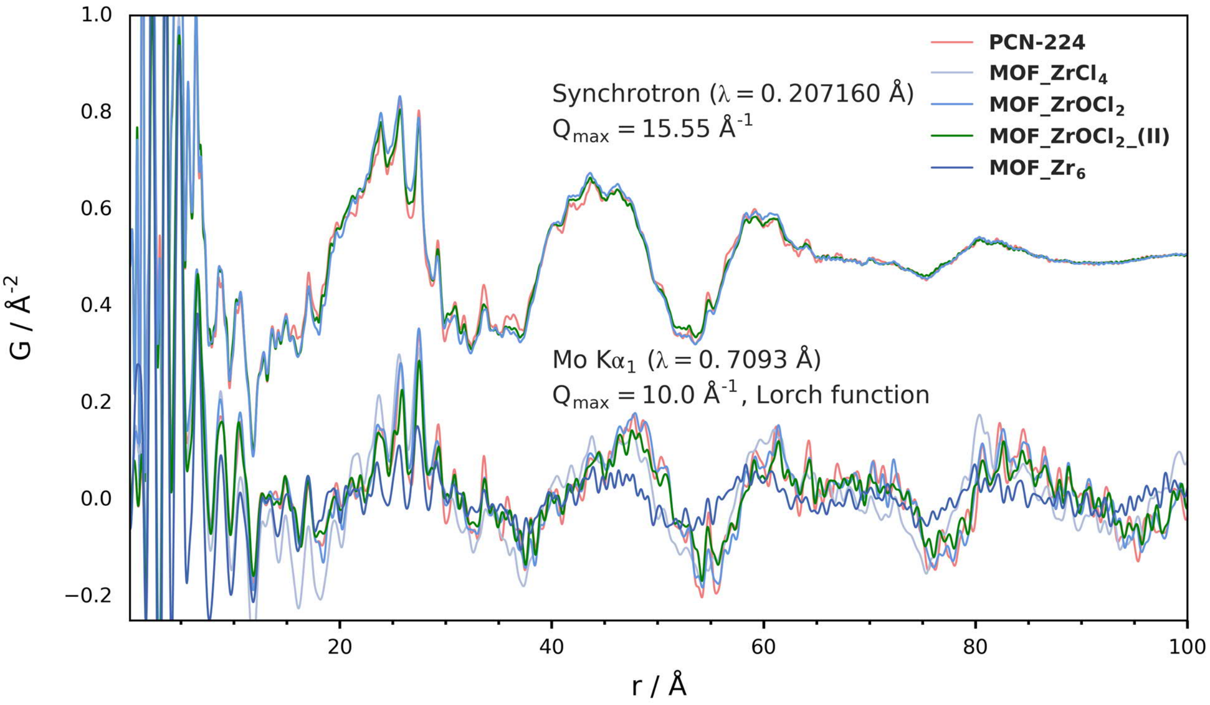

Figure S17: Pair distribution functions $\boldsymbol{G(r)}$ (PDFs) of all samples at long ranges. PDFs shown as obtained from both synchrotron and Mo Kα_1 measurements shown over a longer distance range. There is additionally good agreement between the PDFs obtained for all samples at high distances, indicating that despite differences in the diffraction patterns, the long range structuring is also highly similar.

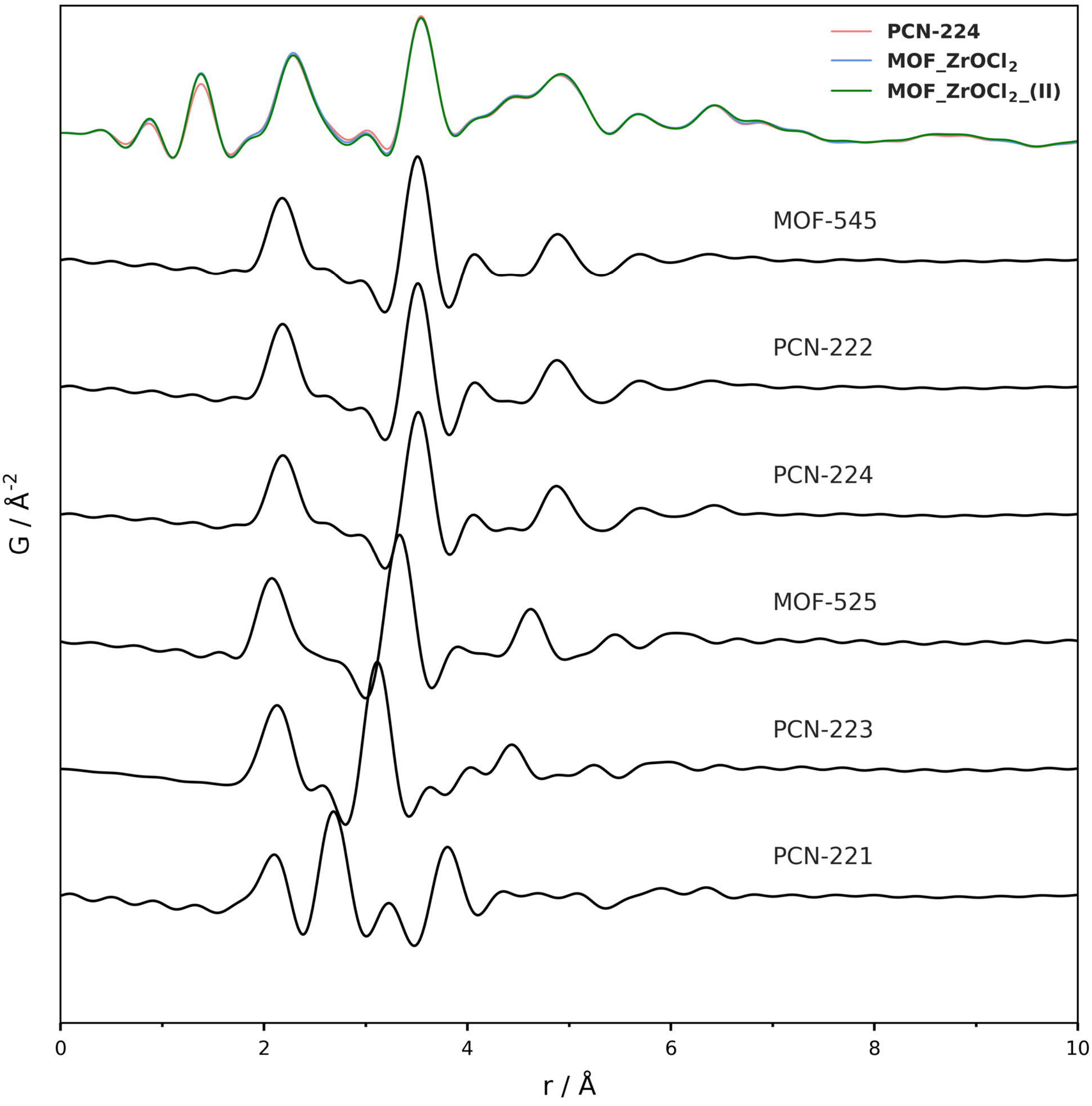

Figure S18: Comparison of the pair distribution functions (PDFs) of samples PCN-224, MOF_ZrOCl$_2$, MOF_ZrOCl$_2$_(II) to PDFs simulated for the ZrO clusters from different crystal structures published for this MOF family. It is clear that the cluster structure is the same for all experimental structures, and matches well to the bond distances for the Zr$_6$ clusters published for MOF-545, PCN-222, and PCN-224. The bond distances for the Zr$_6$ cluster in MOF-525, and especially in PCN-223, are contracted compared to what we observe experimentally, but otherwise the signals match. A completely different bond distance distribution is observed for the Zr$_8$ clusters published for PCN-221, which contains Zr–Zr pair distances not observed experimentally.

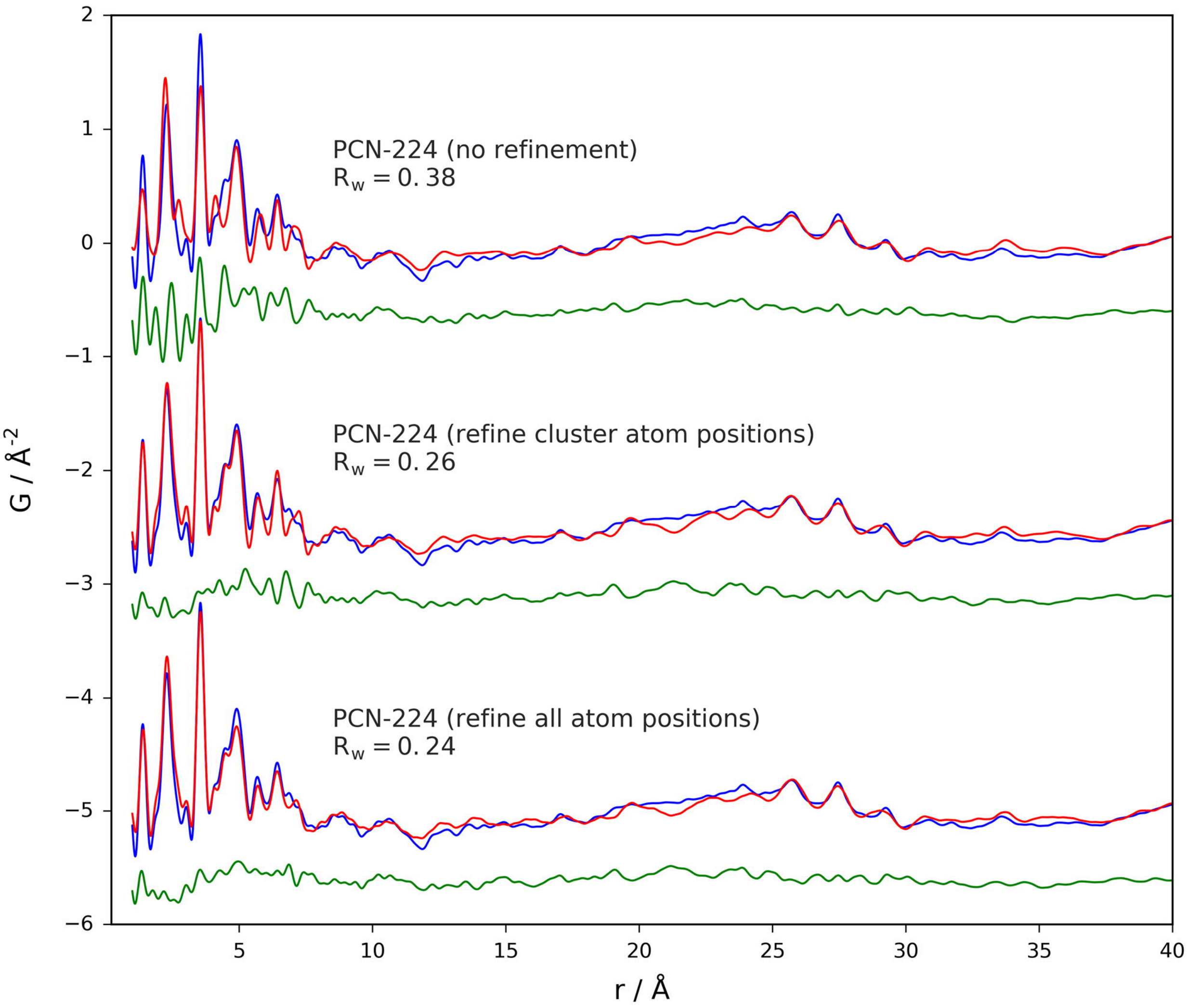

Figure S19: Fits from structure refinement of the published PCN-224 structure to the observed synchrotron pair distribution function of sample PCN-224. Fitting was carried out first by refinement of only the lattice parameter, scale factor, and peak sharpening due to correlated motion. The fit was significantly improved just by refining the Zr and O atom positions within the cluster by symmetry. The best fit came by additionally refining the porphyrin atom positions by symmetry as well, which did not vary substantially from their original positions. The fit gives very good agreement at short distances, and fairly good agreement to the density distribution over longer distances. There are still some differences which may result from differences in the possible twisting of phenyl groups, slight differences in neighboring cluster structure or orientation, additional coordinating water and/or modulator content, or other factors such as pore content and other defects. Blue: experimental, red: simulation, green: difference.

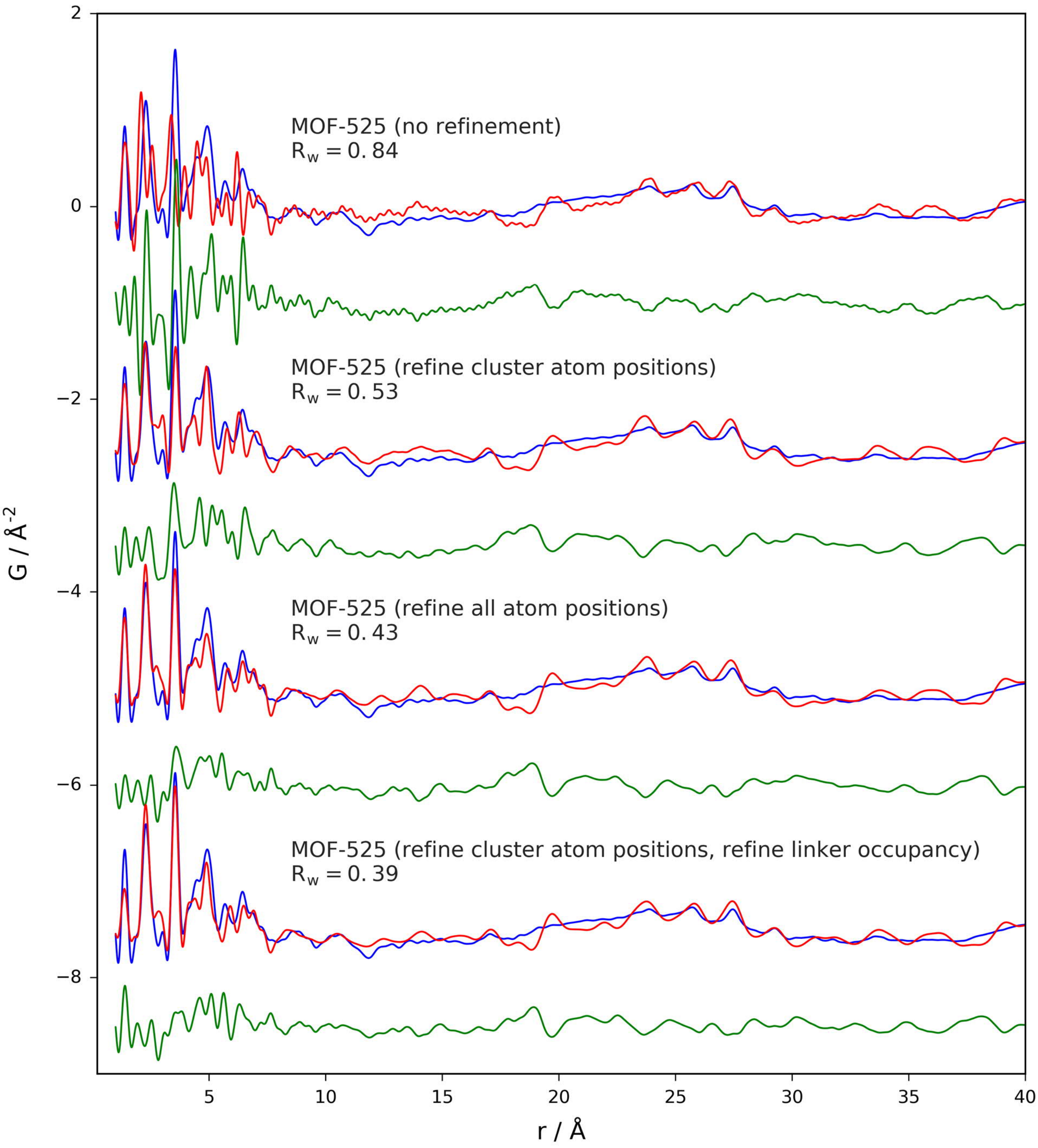

Figure S20: Fits from structure refinement of the published MOF-525 structure to the observed synchrotron pair distribution function (PDF) of sample MOF_ZrOCl$_2$_(II). The first comparison is shown only with sharpened peaks at short distances, and no refinement of lattice parameter or atom positions. As with the Rietveld refinement, the fit is very poor indicating an incorrect structure model. The second fit allowed the lattice parameter and Zr and O positions to refine, and the third fit allowed the porphyrin atoms to refine as well. The fit agreement improved substantially in both cases, but is still very poor. As the density distribution appears to be off in the simulated PDF (e.g. for the long wavelength oscillation of the simulated PDF signal, the trough at approximately 12.5 Å is too shallow), we additionally allowed for there to be occupational vacancies of the linker. An occupation of approximately 68% improved the fit slightly. Blue: experimental, red: simulation, green: difference.

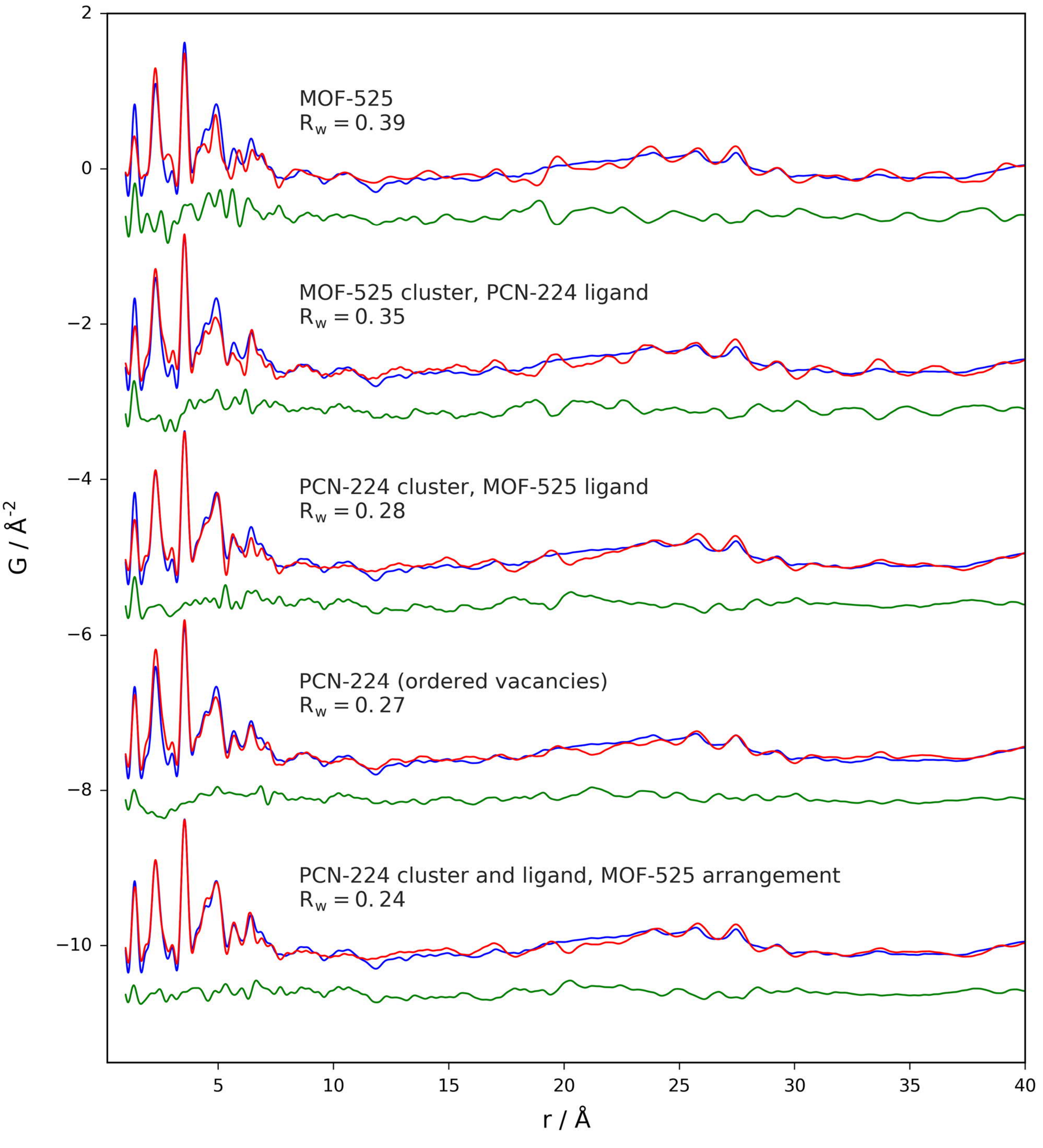

Figure S21: Fits from structure refinement of further modified structures to the observed synchrotron pair distribution function (PDF) of sample MOF_ZrOCl$_2$_(II). The first fit is the last from the previous image, from the MOF-525 model with lattice parameter and atom sites refined along with linker vacancies. In the second fit, the linker was replaced by the conformation found in PCN-224. The third fit instead leaves the flat conformation and replaces the cluster with that found in PCN-224. The fourth fit uses the Im-3m structure published for PCN-224 with ordered vacancies. The last fit uses the MOF-525 structure but with PCN-224 linker and cluster orientation along with refined linker occupation (52%). As with the PDF fits of the experimental PCN-224 sample, some remaining misfit may be due to distributions of twists in the phenyl groups, slight differences in neighboring cluster structure or orientation, additional coordinating water and/or modulator content, or other factors such as pore content and other defects. Blue: experimental, red: simulation, green: difference.

2.5. Single-Crystal X-ray Diffraction

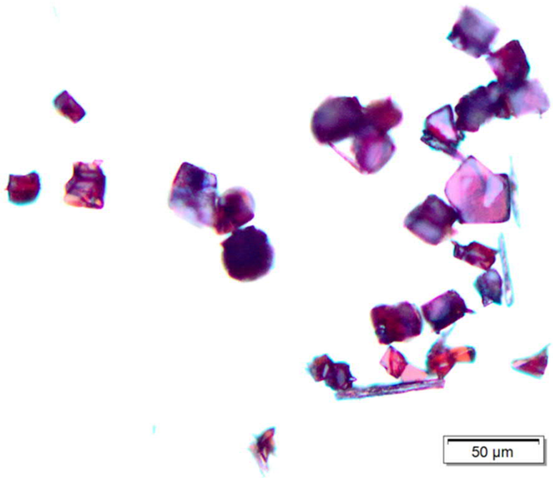

Figure S22: Microscope image of *d*PCN-224 singles crystals. Powder X-ray diffraction pattern of the single crystals can be found in Figure S3 showing no superstructure reflections as in PCN-224.

A purple block-shaped crystal of 0.025 x 0.015 x 0.015 mm^3 size was measured (Figure S22). The diffraction data was indexed with a primitive, cubic unit cell with *a* = 19.315(2) Å and had an internal *R*-value of 34% due to small crystal size and a low scattering intensity of the MOF. The structure solution in several primitive, cubic space groups gave the linker molecule in good completeness and revealed repeatedly a Zr_8O_6 cube with a Zr–Zr distance of 2.55 Å. Accepting these solutions, residual electron density was mainly found around the Zr atom, arranged in the triangle. Based on this, we considered a superposition of Zr_6 octahedra and designed the structural model of four differently oriented Zr_6 clusters with in the cuboctahedron of linker molecules.

This structural model can be described in space group $Pm\bar{3}m$ (no. 221) by only one Zr position (Wyckoff position 24*m*) with an occupancy of 25% for the structure refinement (only one out of four orientations of the Zr_6 clusters can be present). The refinement optimized the Zr position so that the averaged corner-to-corner distance of the truncated cube arrived at 2.70 Å, which agrees perfectly with the originally published edge length of the Zr_8O_6 cluster of 2.69 Å.[30] The Zr–Zr distances are on average 3.49 Å (6×3.457(1) Å and 6×3.529(1) Å) and agree well with the bond length in ZrO_2 (3.45 Å). The disoriented model improved the *R*-value compared to the Zr_8 cube by 12%. Next, we added the O atoms of a typical Zr_6O_8 cluster to our model. O1 is localized in the corner of the truncated cube when there is no Zr present in that corner (occupancy = 25%). O2 is above the faces of the cube (in all the cases, occupancy = 100%). The O atoms of the carboxylate group could be found by residual electron density around the carboxylate carbon atom in two different orientations (O3 and O4, occupancy = 0.5×phenyl ring occupancy = 38%) that are necessary to bridge and chelate the Zr atoms of the Zr_6 clusters (Figure S23). By adding the O atoms to

our model the *R*-value improved by 10%. Further, Hydrogen atoms, bonded to carbon atoms, were added to the structure model on calculated positions using a riding model. Isotropic displacement parameters were set to $1.2\times U_{eq}$ of the attached carbon atom We divided the linker in two group to refine its occupancy, namely the porphyrin ring (N, C1–C3, H2) and the phenyl ring (C4–C8, H6, H7, O3, O4). The occupancy of the porphyrin ring (refined to 50%) represents the actual linker molecule while the occupancy of the phenyl ring (refined to 75%) is the sum of linker and modulator molecule of the same structure (benzoate). We assume the latter occupancy deviates from 100% due to the enormous degree of freedom of the benzoate. We have refrained from refining C and N atoms with anisotropic ADP but we were able to refine them individually. The ADPs of the porphyrin ring atoms are smaller than the ADPs of the phenyl ring atoms. The largest ADPs are found for C6 and C7 pointing towards a porphyrin–phenyl dihedral angle deviating locally from 90°. In the end, low residual electron density is located only in the middle of the pores (0.770/–0.496 e $Å^{-3}$) and may be attributed to solvent and modulator molecules left in the porous material. We have refrained from manipulating our raw diffraction data to subtract this small value for a small *R*-value reduction. Furthermore, we have refrained from refining O, C, and N atoms with anisotropic displacement parameters. The refinement arrived at an R_1-value of 17% ($I > 2\sigma(I)$) or 26% (all data) which is high in general for a single-crystal refinement but agreeable for a MOF due to small crystallite size, low intensity of the collected data, and the fast decay of the intensity to high diffraction angles.

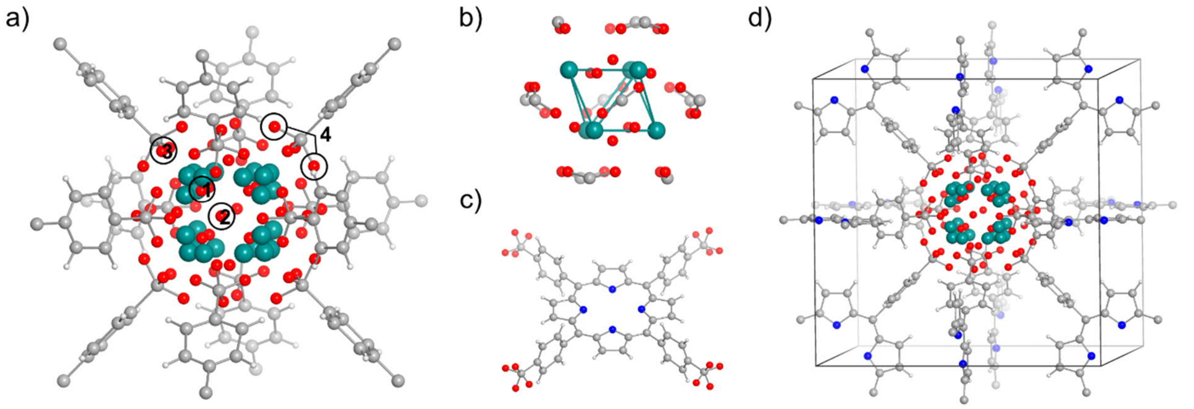

Figure S23: Structure model obtained from single crystal X-ray diffraction (SCXRD). a) SCXRD refined oxygen positions 1–4 (red) in the disordered model. Please refer to text for explanation and note that O3 and O4 might result from coordination with benzoic acid as well. b) Oxygen environment for an individual Zr_6 cluster (teal) with bridging and chelating linker molecules, indicated by the carboxylate groups. c) Tetrakis(4-carboxyphenyl) porphyrin linker obtained from SCXRD. d) Unit cell of the disordered MOF.

The orientation of the Zr_6 cluster in the presented *d*PCN-224 model (corners pointing towards the triangles of the surrounding cuboctahedron of linker molecules) resembles the orientation of the ordered clusters in PCN-224.[37] For this reason we checked the diffraction data for superstructure reflections of a doubled unit cell as in PCN-224 and found indeed *extremely* weak reflections in the *hk*1/2*l* plane for *h* = ±1/2 and *l* = –1/2 (*Figure S24*). These diffuse features imply different local vacancy–orientation environments in the *disordered* single crystal.

MOF-525 differs from both *d*PCN-224 and PCN-224 such that the Zr_6 cluster point toward the squares built by the linker molecules. We tested for the MOF-525 cluster orientation[31] in our single-crystal data and found only overlapping positions ($Zr_{MOF-525}$ on O2 and $O_{MOF-525}$ on O1). Kinetic limitations and configurational entropy are discussed in the publications.

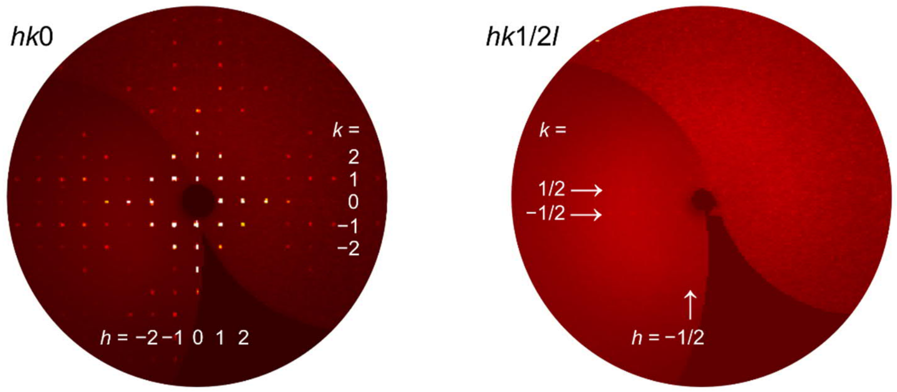

Figure S24: Single-crystal X-ray diffraction. Data presented for the hk0 and hk1/2l scattering planes.

A few words on the limitations of this single-crystal structure compared to the local structure: The solution in space group $Pm\bar{3}m$ (no. 221) restricts the linker molecule to be flat with a 90° dihedral angle between the porphyrin and phenyl ring as well as 0° and 90° dihedral angles between the phenyl ring and carboxylate group. Instead, we conclude from ADP sizes that the linker are more flexible to adapt the local structure as in PCN-224 with porphyrin–phenyl and phenyl–carboxylate dihedral angles of 78° and 4°, respectively.[37] However, single-crystal diffraction detects long-range order and displays average electron density. For details on the local structure, linker defects, and the coordination with benzoic acid refer to results of the local probes PDF, Rietveld, NMR, and DFT in this publication.

Table S6: Crystallographic data of *d*PCN-224 as obtained from single crystal X-ray diffraction.

Formula	$Zr_6\,O_{26}\,N_6\,C_{91}\,H_{42}$
Crystal shape	block, 0.025 x 0.015 x 0.015 mm^3
Crystal color	purple transparent
Radiation	Cu $K\alpha$
Temperature T, K	100
Crystal symmetry	cubic
Space group	$Pm\bar{3}m$ (no. 221)
Lattice parameter a, Å	19.315(2)
Cell volume V, $Å^3$	7206(2)
Cell constant Z	1
Density $d_{calc.}$, g/cm^3	0.517
Absorption coefficient μ, mm^{-1}	1.931
Structure factor $F(000)$	1112
2θ range, °	$2.287 \leq 2\theta \leq 58.972$
Index range	$-21 \leq h \leq 17$
	$-21 \leq h \leq 18$
	$-21 \leq h \leq 18$
Reflections collected	28398
Independent reflections	1106 (R_{int} = 0.3368)
Completeness to theta = 58.972°	100.0%
Refinement method	Full-matrix least-squares on F^2
Data / restraints / parameters	1106 / 0 / 34
R_1/wR_2 ($I > 2\sigma(I)$)	0.178/0.408
R_1/wR_2 (all data)	0.262/0.436
GOF on F^2	2.23
$\Delta\rho_{max}/\Delta\rho_{min}$ (max/min), e $Å^{-3}$	0.770/–0.496

Table S7: Atomic coordinates and equivalent isotropic displacement factors U_{eq} (Å^2) for *d*PCN-224 as obtained from single crystal X-ray diffraction at T = 100 K.

atom	site	*x*/*a*	*y*/*b*	*z*/*c*	U_{eq}	S.O.F.
Zr1	24*m*	0.5447(2)	0.5847(2)	0.5847(2)	0.110(4)§	0.25
O1	8*g*	0.5530(20)	0.5530(20)	0.5530(20)	0.070(30)	0.25
O2	6*f*	1/2	0.6166(19)	1/2	0.255(16)	1.00
O3	24*m*	0.5551(13)	0.6403(9)	0.6403(9)	0.114(9)	0.375(8)
O4	24*l*	1/2	0.6906(15)	0.6034(16)	0.154(12)	0.375(8)
N1	12*h*	1/2	1	0.8922(15)	0.095(11)	0.471(17)
C1	24*l*	1/2	0.9412(13)	0.8512(13)	0.094(10)	0.471(17)
C2	24*l*	1/2	0.9617(13)	0.7811(15)	0.108(11)	0.471(17)
C3	12*j*	1/2	0.8734(13)	0.8734(13)	0.085(12)	0.471(17)
C4	12*j*	1/2	0.8186(12)	0.8186(12)	0.140(12)	0.749(16)
C5	12*j*	1/2	0.7217(14)	0.7217(14)	0.165(14)	0.749(16)
C6	24*m*	0.5621(16)	0.7411(12)	0.7411(12)	0.221(14)	0.749(16)
C7	24*m*	0.5569(15)	0.7976(12)	0.7976(12)	0.213(14)	0.749(16)
C8	12*j*	1/2	0.6616(15)	0.6616(15)	0.166(15)	0.749(16)
H2Δ	24*l*	1/2	0.9323	0.7417	0.130	0.471(17)
H6Δ	24*m*	0.6041	0.7230	0.72367230	0.266	0.749(16)
H7Δ	24*m*	0.5981	0.8165	0.8165	0.256	0.749(16)

§ Anisotropic displacement factor: 0.116(5) 0.103(5) 0.103(5) 0.059(4) –0.064(3) –0.064(3).

Δ Calculated.

2.6. Solid-state NMR spectroscopy

High-resolution ^{1}H, ^{13}C and ^{15}N magic angle spinning (MAS) NMR spectra of *d*PCN-224 and ordered PCN-224 (Figures 6, S25-S27) are intriguingly similar, supporting the proposed structural relation between both compounds on local length scales. The NMR spectra show well resolved resonances for all characteristic chemical groups of the TCPP linkers, the modulator molecules (acetate (Ac) and benzoate (Bz)) and the Zr clusters. In particular, the presence of sharp and well-defined resonances for the µ3-OH groups at around 3.3 ppm in the ^{1}H MAS NMR spectra (Figures 6a and S25) underlines the presence of $Zr_6O_4(OH)_4$ clusters, which is in line with both the diffraction and PDF data. Additional signals between 0 and 6 ppm were observed which, however, do not show double quantum (DQ) correlations (Figure S28). This behavior is characteristic for hydrogen atoms of mobile hydroxy groups or water molecules, not involved in hydrogen bonding. The ratio between the intensities of the resonances of the µ3-OH (≈ 3.3 ppm) and the NH units (≈ –3.2 ppm) of the TCPP linkers provides a straightforward estimate for the linker to cluster proportion for both *d*PCN-224 and experimental PCN-224. While on average 1.6(1) TCPP molecules complement one Zr cluster for *d*PCN-224, the value is somewhat lower for ordered PCN-224 with 1.2(1). Compared to 12-fold coordination of the $Zr_6O_4(OH)_4$ clusters, this amounts to 53(4)% (*d*PCN-224) and 40(4)% (experimental PCN-224) linker occupancy and thus to an average TCPP coordination number of the cluster of roughly 6 and 5, respectively. The lower coordination density for $Zr_6O_4(OH)_4$ for *d*PCN-224 and PCN-224 explains the lowfield shift of about 1 ppm for the µ3-OH groups with respect to the one observed for UiO-67.[42]

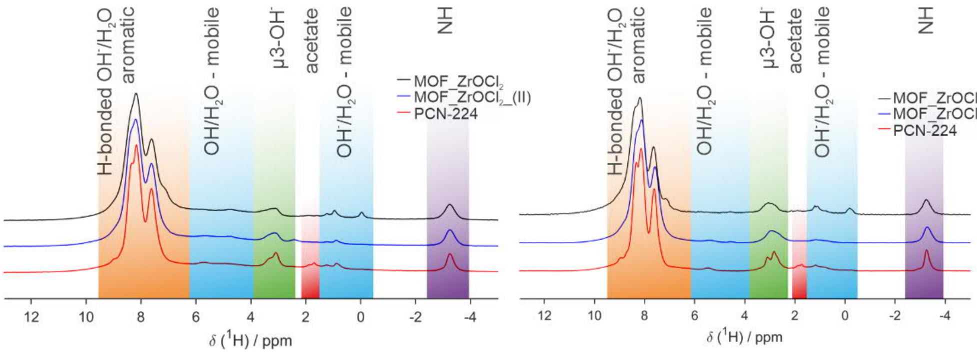

Figure S25: High-resolution ^{1}H magic angle spinning (MAS) NMR. Spectra of MOF_ZrOCl₂ (black), MOF_ZrOCl₂_(II) (blue), and experimental PCN-224 (red). Left: ^{1}H spin echo spectra recorded at a Lamor frequency of 600 MHz. Right: ^{1}H single pulse spectra acquired at a Lamor frequency of 1 GHz. The difference for both data sets within region color-coded in blue is due to the faster spin-spin relaxation of mobile water molecules and hydroxy groups assigned to these regions. The results of deconvolution of the ^{1}H single pulse (SP) spectra (ν_0 = 1 GHz), with pseudo-Voigt (pVoigt) profiles is given in tables S8 – S10.

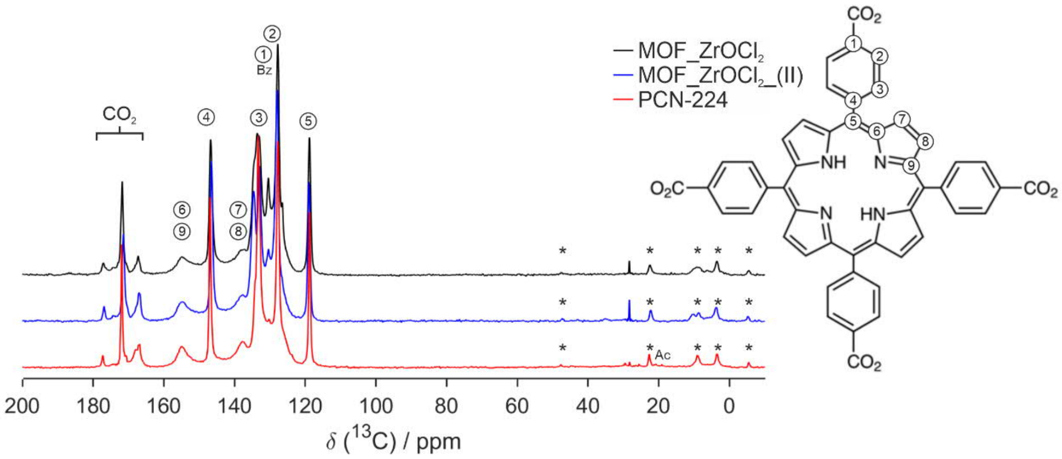

Figure S26: ^{13}C cross polarization magic angle spinning (CPMAS) NMR. Spectra of MOF_ZrOCl$_2$ (black), MOF_ZrOCl$_2$_(II) (blue), and experimental PCN-224 (red). All resonances are assigned to the tetrakis(4-carboxyphenyl) porphyrin linkers, benzoate (Bz), and acetate (Ac). Spinning sidebands are marked by an asterisk.

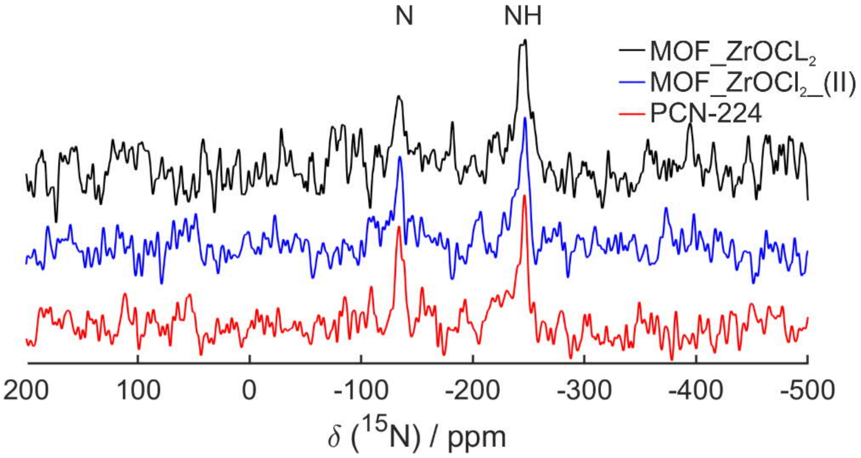

Figure S27: ^{15}N cross polarization magic angle spinning (CPMAS) NMR. Spectra of MOF_ZrOCl$_2$ (black), MOF_ZrOCl$_2$_(II) (blue), and experimental PCN-224 (red), with the characteristic resonances for the tertiary (N) and secondary (NH) nitrogen atoms of the porphyrin rings of the tetrakis(4-carboxyphenyl) porphyrin linkers.

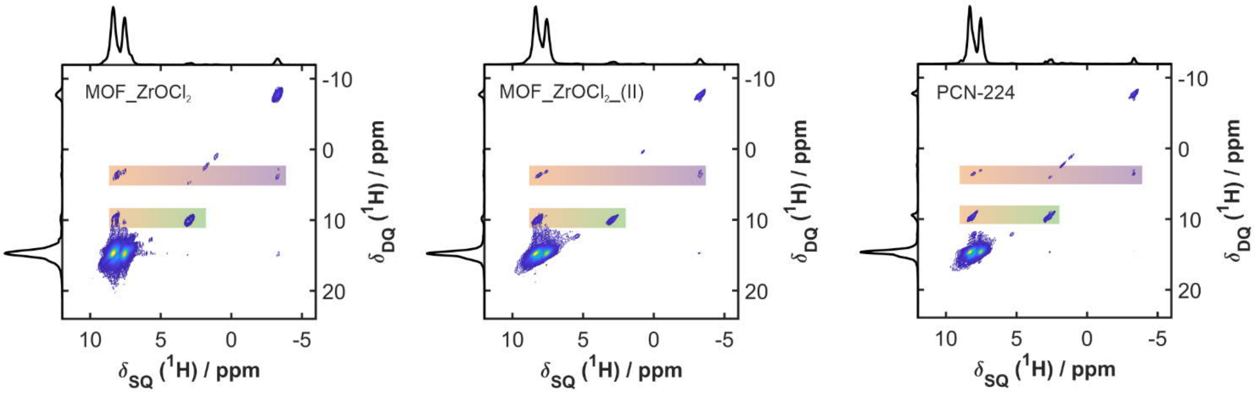

Figure S28: 1H-1H double-quantum single-quantum (DQSQ) NMR. Spectra of the *d*PCN-224 variants MOF_ZrOCl$_2$ (left) and MOF_ZrOCl2_(II) (middle), and experimental PCN-224 (right). Cross correlations show proximities of aromatic protons to NH protons of the the tetrakis(4-carboxyphenyl) porphyrin linkers (ν_{DQ} = 3.8 ppm, color-coded orange to purple), and aromatic protons to µ3-hydroxyl protons (ν_{DQ} = 9.7 ppm, color-coded orange to green).

Table S8: Refinement parameters of the deconvolution of the $^{1}H(\nu_0$ = 1 GHz) SP spectrum of experimental PCN-224 with pVoigt profiles. G/L describes the ratio between Gaussian and Lorentzian part of the pVoigt profile, where the boundary values 1 and 0 represent pure Gaussian and Lorentzian functions, respectively. Color-codes are associated to assignments in Figure 6a and S25.

δ_{iso} / ppm	*G/L*	*FWHM* / ppm	*Integral* / arb. units
–3.27	0.26	0.21	2.00
1.29	1.00	1.41	2.01
1.81	1.00	0.25	0.19
2.70	0.20	0.35	1.33
2.84	0.20	0.19	1.06
3.10	0.20	0.22	1.09
5.50	0.28	0.37	0.35
7.61	0.23	0.28	9.47
8.11	0.30	0.29	12.61
8.36	0.30	0.27	9.53
8.92	0.00	0.79	2.52

Table S9: Refinement parameters of the ^{1}H(ν_0 = 1 GHz) SP spectrum of MOF_ZrOCl$_2$ fitted with pVoigt profiles. Color-codes are associated to assignments in Figure 6a and S25.

δ_{iso} / ppm	*G/L*	*FWHM* / ppm	*Integral* / arb. units
–3.25	0.74	0.38	2.00
–0.19	0.58	0.26	0.59
0.97	0.00	0.65	1.35
1.20	1.00	0.23	0.29
1.80	1.00	1.00	0.96
1.94	0.25	0.01	0.01
3.01	0.90	0.69	2.59
4.10	0.80	0.80	0.54
5.11	0.80	1.00	0.60
6.30	0.80	1.00	0.79
7.14	0.80	0.36	1.32
7.64	0.50	0.33	9.25
8.13	0.80	0.33	9.81
8.42	0.80	0.40	11.88
9.10	0.70	1.00	5.04
8.80	0.80	0.38	1.62

Table S10: Refinement parameters of the ^{1}H(ν_0 = 1 GHz) SP spectrum of MOF_ZrOCl$_2$_(II) fitted with pVoigt profiles. Color-codes are associated to assignments in Figure 6a and S25.

δ_{iso} / ppm	*G/L*	*FWHM* / ppm	*Integral* / arb. units
–3.29	0.69	0.38	2.00
0.80	1.00	0.55	0.28
1.17	1.00	0.39	0.21
1.78	0.80	0.97	0.52
2.68	1.00	0.59	1.06
2.99	0.80	0.52	1.38
4.55	1.00	0.12	0.01
5.80	0.00	1.00	0.96
7.58	0.70	0.47	11.12
8.10	0.80	0.36	12.13
8.38	0.70	0.32	7.66
8.80	0.70	0.82	4.32

The intensity of the ^{1}H NMR resonances for the aromatic protons of the TCPP linkers (7–9 ppm) is coupled to the ones of the NH units (≈ –3.2 ppm). For one NH proton, 12 protons in the aromatic region are expected. However, for all *d*PCN-224 samples and the reference PCN-224, a ratio between 1:17 and 1:18 was determined, which requires either additional components with aromatic, or low-field shifted protons as for e. g. reported for hydroxy water pairs saturating the coordinatively unsaturated sites (CUS) of the $Zr_6O_4(OH)_4$ clusters in NU-1000.[43] As experimental PCN-224 was synthesized using acetic acid, only the second scenario is possible for this compound. The intensity of the methyl protons of residual acetate ions (Figures 6a and S25; ≈ 1.9 ppm) is small and amounts to maximal 0.4(2) acetate ions and five to six hydroxyl water pairs attached to one $Zr_6O_4(OH)_4$ cluster on average. From the chemical shift within the aromatic region (7–9 ppm) of the latter, an O–O distance between 2.6 and 2.7 Å results (Figure 6c), characteristic for strong hydrogen bonds.[44–46] As the spectral line shapes for the ^{1}H and the ^{13}C MAS NMR spectra are very similar for *d*PCN-224 and PCN-224 (Figures 6a, 6b, S25 and S26) and only weak resonances characteristic solely of benzoate (130–140 ppm) were observed (about 0.5(2) Bz on average per cluster (Figure S29)), we expect a similar situation to hold for *d*PCN-224.

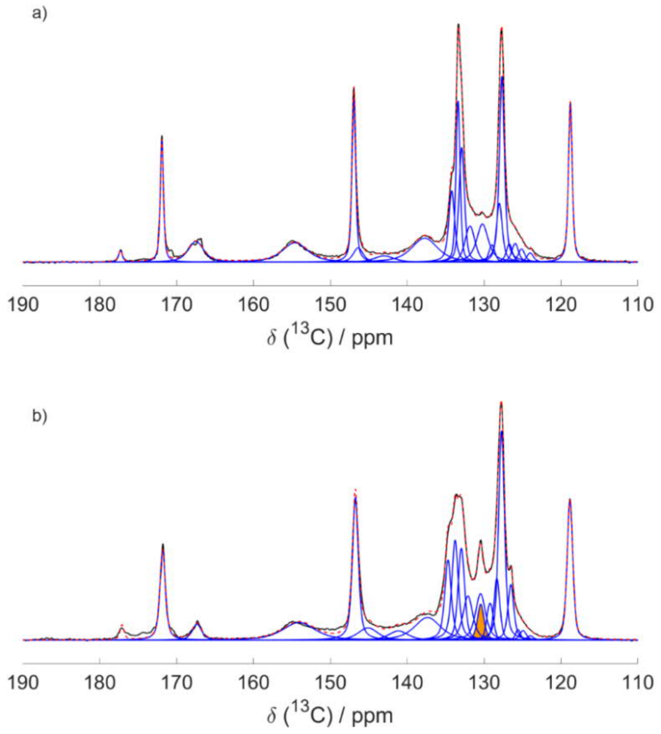

Figure S29: ^{13}C cross polarization magic angle spinning (CPMAS) NMR deconvoluted with pseudo-Voigt profiles (blue). Spectra of a) experimental PCN-224 and b) MOF_$ZrOCl_2$. The additional signal (colored in orange) at ~130 ppm in (b) is assigned solely to the ortho- and para-CH units of benzoic acid and is used for an estimation of modulator/linker ratio. The ipso carbon and meta-CH groups are superimposed with the ones of tetrakis(4-carboxyphenyl) porphyrin (TCPP). Integration leads to roughly 0.31(1) benzoate ions per TCPP linker and thus to 0.5(2) benzoate ions per cluster.

The most telling region within the ^{13}C MAS NMR spectra for both *d*PCN-224 and PCN-224 is the carboxylic region (165–180 ppm), which contains several signals with maxima around 167.3 and 177.2 ppm centered around a main peak at ≈ 171.9 ppm (Figures 6b and S26). The broad spread observed for the shifts of the carboxylate resonances is indicative of different binding modes between the $Zr_6O_4(OH)_4$ clusters and the carboxylate units of the TCPP linkers combined with a configurational variance on the cluster. DFT calculations for $[Zr_6O_4(OH)_4]Ac_{12}$ clusters (Figure S30) with configurations that contain Ac ions in bridging coordination only and mixed bridging as well as chelating Ac ions demonstrate, in line with ref. [47], that bridging carboxy groups are high-field shifted by 6 – 10 ppm with respect to chelating ones, depending on the cluster configuration. On average, and again in agreement with ref. [47] chelating carboxylate groups are less favored by 15–16 kJ/mol. In addition, the majority of the carboxylate groups of the TCPP linkers in PCN-224 are reported to coordinate in a bridging mode.[32] Hence, we assign the central resonance and its high-field region to carboxylates in a bridging motif (Figure 6c) and the low-field region (172–176 ppm) to the chelating binding motif (Figure 6c). As such, the majority of carboxylate groups is coordinated in a bridging mode for *d*PCN-224 as well, which allowed to refine the structure model derived from the scattering data further. The broad distribution for the resonances of the µ3-OH groups (≈ 3.3 ppm; Figure 6a and S25), also points towards a configurational variance of bridging and chelating binding motifs, as well as of hydroxy water pairs on the cluster.[48]

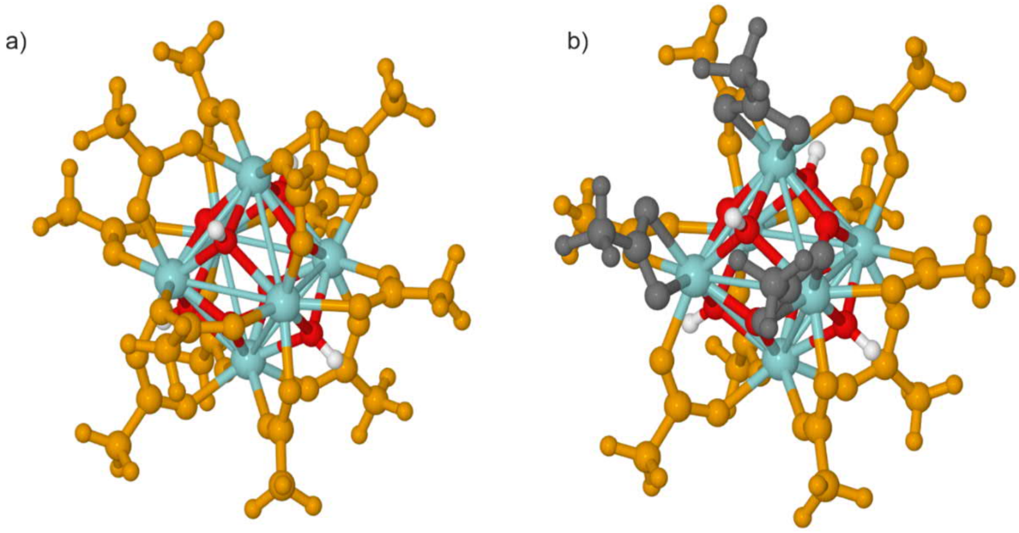

Figure S30: DFT optimized $[Zr_6O_4(OH)_4]Ac_{12}$ clusters. Optimization with a) only bridging carboxylates and b) with mixed linker environment, where three acetates chelate three Zr atoms of one µ3-OH capped face of the Zr_6 octahedron. The bridging acetates are highlighted in ocher, while the chelating acetates are color-coded in grey. The cluster with mixed linker environment is 0.4855 eV higher in energy.

The distribution of different binding modes is also reflected in the lineshapes of the ^{91}Zr wideline NMR spectra of *d*PCN-224 and ordered PCN-224 (Figure 6d) recorded using the variable offset cumulative spectra (VOCS) approach combined with a quadrupolar Carr-Purcell-Meiboom-Gill (qCPMG) sequence.[13–15,49] Both ^{91}Zr NMR spectra consist of at least four different quadrupolar shapes with non-axial symmetric coupling tensors and coupling constants (C_Q) between 10 and 25 MHz. This proves a markedly more diverse electronic situation for the individual Zr atoms compared to the fully saturated $Zr_6O_4(OH)_4Bz_{12}$[50] used as starting material in one of the *d*PCN-224 syntheses, for which two superimposing quadrupolar lineshapes with coupling constants C_Q of about 14 and 20 MHz were observed (Figure S31). According to the DFT calculations (Figure S30) Zr atoms exclusively coordinated by bridging carboxylates exhibit the larger C_Q values compared to those involved in mixed binding modes. The broader variance for *d*PCN-224 and ordered PCN-224 reflects the disorder induced by the hydroxyl ions and water molecules coordinating to the CUS and the strain on the Zr clusters imposed by the framework topology.

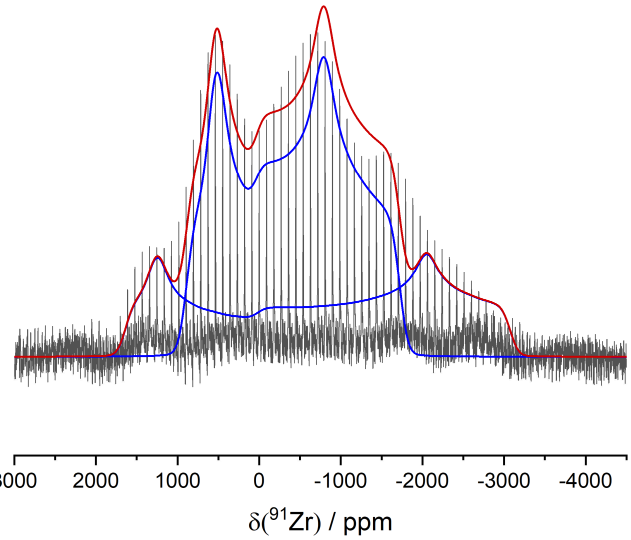

Figure S31: ^{91}Zr qCPMG NMR VOCS of $Zr_6O_4(OH)_4Bz_{12}$ deconvoluted into two quadrupolar shapes (blue). This is in line with the structure of the cluster proposed in ref. [50] The structure solution consists of 3 chelating and 9 bridging Bz, resulting in two Zr environments. One where only bridging carboxylate groups coordinate and the other where bridging and chelating carboxylate units are mixed.

Table S11: Refinement parameters of the deconvolution of ^{91}Zr NMR spectrum (Figure S30) of $[Zr_6O_4(OH)_4]Bz_{12}$ with two second order quadrupolar lineshapes. δ_{iso}: isotropic chemical shift, C_Q: quadrupolar coupling constant, η_Q: Anistropy of quadrupolar coupling tensor, LB: Lorenzian line broading, GB: Gaussian line broadening.

	1	2
δ_{iso} / ppm	0	0
C_Q / MHz	14	20
η_Q	0.4	0.2
LB / Hz	1000	1000
GB / Hz	10000	10000
Integral /arb. units	0.65	0.35

Table S12: Refinement parameters of the deconvolution of ^{91}Zr NMR spectrum (Figure 6d) of *d*PCN-224 with four second order quadrupolar lineshapes.

	1	2	3	4
δ_{iso} / ppm	408	–142	–780	–600
C_Q / MHz	21	19	23	18.5
η_Q	0.5	0.3	1.0	0.6
LB / Hz	1000	1000	1000	1000
GB / Hz	10000	10000	10000	10000
Integral /arb. units	0.31	0.29	0.25	0.15

Table S13: Refinement parameters of the deconvolution of ^{91}Zr NMR spectrum (Figure 6d) of experimental PCN-224 with four second order quadrupolar lineshapes.

	1	2	3	4
δ_{iso} / ppm	622	–1850	285	–4000
C_Q / MHz	24	14	15	13
η_Q	0.7	1.0	0.0	0.1
LB / Hz	1000	1000	1000	1000
GB / Hz	10000	10000	10000	10000
Integral /arb. units	0.33	0.33	0.24	0.10

2.7. Quantum chemical calculations

Zr_8O_6 Cluster

The structure of a Zr_8O_6-Cluster was extracted from the published crystal structure for PCN-221[30]. Using this structure as an initial starting point, the geometry was optimized on PBE0-D3/def2-mSVP[23–26] level of theory. Zr–Zr distances within the initial Zr_8O_6 cluster reside at 2.688 Å, 3.802 Å and 4.656 Å as Zr–Zr distances to the closest, face diagonal and space diagonal neighbor.

All distances expand during the optimization significantly to 6.938 Å, 9.811 Å and 12.016 Å as Zr–Zr distances to the closest, face diagonal and space diagonal neighbor, beyond the distance of bound Zr. These pre-converged results indicate, that the distances within the initial structure of a Zr_8O_6-Cluster are unfeasible.

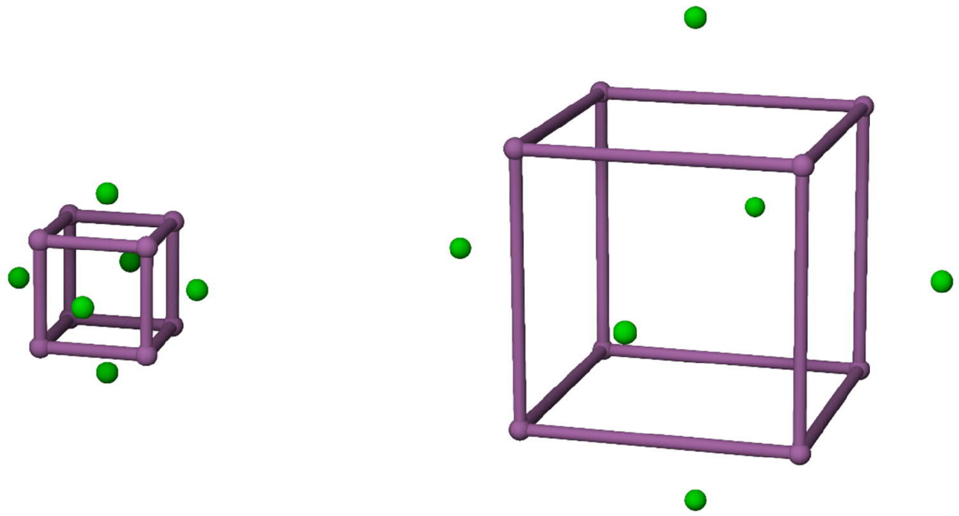

Figure S32: Structure of the Zr_8O_6 cluster. Left: Initial structure, extracted from the published crystal structure of PCN-221[30]; Right: Pre-Converged structure, obtained on PBE0-D3/def2-mSVP level of theory.

Zr$_6$O$_8$ Cluster

The comparison of the optimized Zr$_6$O$_8$ cluster obtained on PBE0-D3/def2-mSVP level of theory with the Zr$_6$O$_8$ cluster obtained from total scattering pair distribution function (PDF) analysis of *d*PCN-224, shows a good agreement on the position of the zirconium atoms, whereas the position of the modeled oxygen atoms deviate.

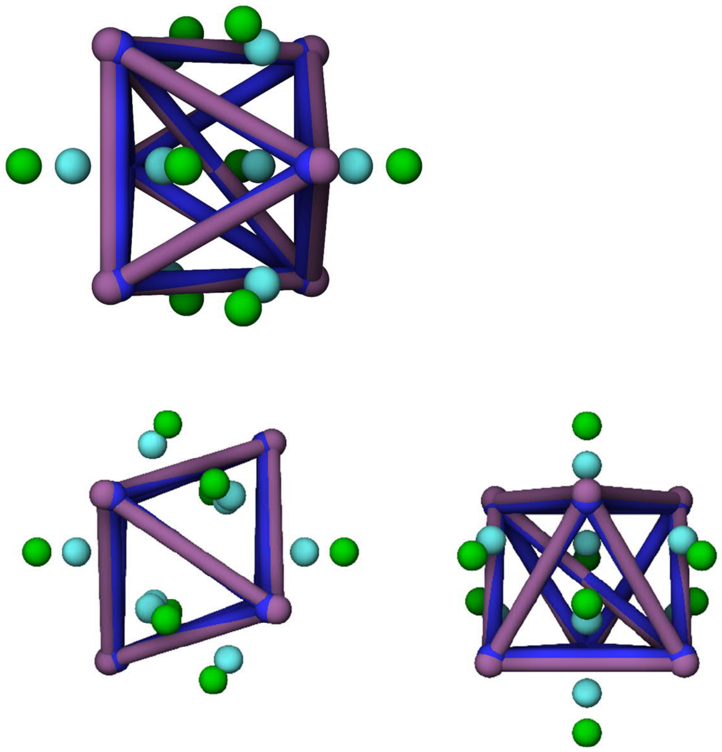

Figure S33: Overlay of the optimized Zr$_6$O$_8$ cluster and cluster structure obtained from total scattering pair distribution function (PDF). The optimized Zr$_6$O$_8$ cluster obtained on PBE0-D3/def2-mSVP level of theory is depicted in purple (Zr$_6$ octahedron) and light blue (oxygen). The Zr$_6$O$_8$ cluster structure obtained from (PDF) analysis of *d*PCN-224 is depicted in blue (Zr$_6$ octahedron) and green (oxygen).

Rotation profile of the TCPP linker

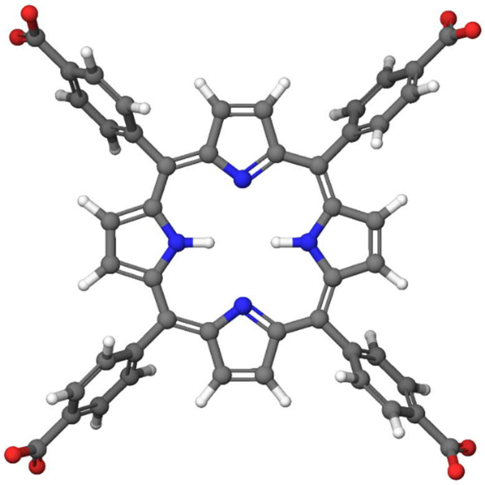

Figure S34: Optimized geometry of the tetrakis(4-carboxyphenyl) porphyrin linker. Optimized structure obtained on PBE0-D3/def2-TZVP level of theory.

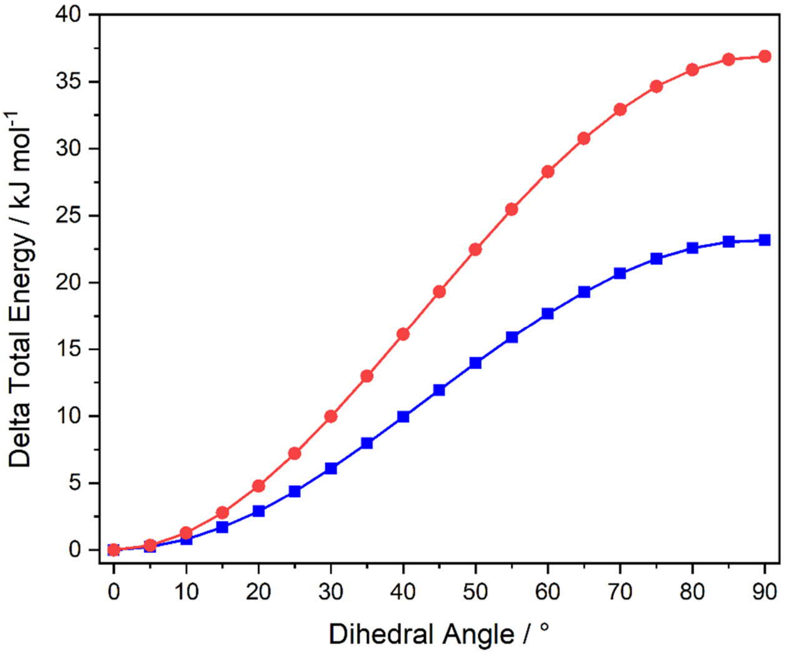

Figure S35: Rotation profile of the phenyl–carboxylate (PHE-CAR) dihedral angle in tetrakis(4-carboxyphenyl) porphyrin (TCPP). Rotation profile within the optimized geometry of the TCPP linker, obtained on PBE0-D3/def2-TZVP//PBE0-D3/def2-TZVP (blue) and PBE0-D3/def2-mSVP//PBE0-D3/def2-mSVP (red) level of theory. The minimum indicates a preferred dihedral angle of 0°.

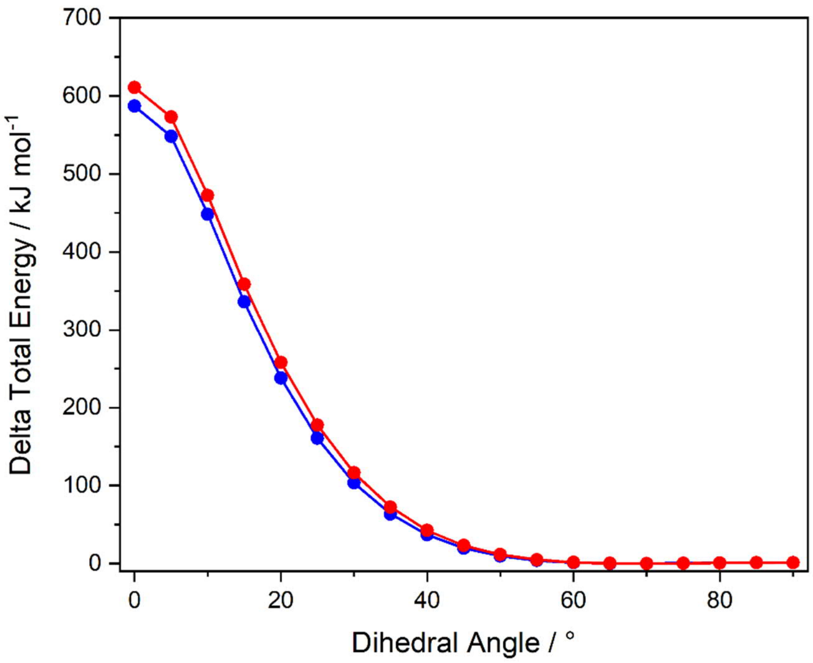

Figure S36: Rotation profile of the porphyrine–phenyl (POR-PHE) dihedral angle in tetrakis(4-carboxyphenyl) porphyrin (TCPP). Rotation profile within the optimized geometry of the TCPP linker, obtained on PBE0-D3/def2-TZVP//PBE0-D3/def2-TZVP (blue) and PBE0-D3/def2-mSVP//PBE0-D3/def2-mSVP (red) level of theory. The maximum of ~600 kJ/mol at 0° clearly visualizes the preferred planarity of the POR-PHE dihedral angle.

Table S14: Energies of the rotation of the phenyl–carboxylate (PHE-CAR) dihedral angle of the tetrakis(4-carboxyphenyl) porphyrin linker. The decrease of favorable interactions with increasing dihedral angle is highlighted with a color gradient from green (most favored) to red (least favored).

	PBE0-D3/def2-TZVP			PBE0-D3/def2-mSVP		
Dihedral Angle [°]	Total Energy [H]	Delta Total Energy [H]	Delta Total Energy [kJ/mol]	Total Energy [H]	Delta Total Energy [H]	Delta Total Energy [kJ/mol]
0	-2663.759709	0.000000	0.0	-2662.548636	0.000000	0.0
5	-2663.759623	0.000086	0.2	-2662.548508	0.000128	0.3
10	-2663.759409	0.000300	0.8	-2662.548152	0.000485	1.3
15	-2663.759066	0.000642	1.7	-2662.547581	0.001055	2.8
20	-2663.758608	0.001101	2.9	-2662.546819	0.001818	4.8
25	-2663.758043	0.001665	4.4	-2662.545893	0.002743	7.2
30	-2663.757394	0.002315	6.1	-2662.544840	0.003797	10.0
35	-2663.756679	0.003030	8.0	-2662.543694	0.004943	13.0
40	-2663.755923	0.003785	9.9	-2662.542494	0.006143	16.1
45	-2663.755153	0.004555	12.0	-2662.541276	0.007361	19.3
50	-2663.754389	0.005320	14.0	-2662.540077	0.008560	22.5
55	-2663.753657	0.006051	15.9	-2662.538932	0.009705	25.5
60	-2663.752972	0.006736	17.7	-2662.537868	0.010768	28.3
65	-2663.752360	0.007348	19.3	-2662.536917	0.011719	30.8
70	-2663.751837	0.007872	20.7	-2662.536100	0.012536	32.9
75	-2663.751413	0.008296	21.8	-2662.535444	0.013192	34.6
80	-2663.751109	0.008600	22.6	-2662.534965	0.013672	35.9
85	-2663.750930	0.008778	23.0	-2662.534676	0.013961	36.7
90	-2663.750885	0.008823	23.2	-2662.534589	0.014047	36.9

Table S15: Energies of the rotation of the porphyrine–phenyl (POR-PHE) dihedral angle of the tetrakis(4-carboxyphenyl) porphyrin linker. The increase of favorable interactions with increasing dihedral angle is highlighted with a color gradient from red (least favored) to green (most favored).

Dihedral Angle [°]	PBE0-D3/def2-TZVP Total Energy [H]	Delta Total Energy [H]	Delta Total Energy [kJ/mol]	PBE0-D3/def2-mSVP Total Energy [H]	Delta Total Energy [H]	Delta Total Energy [kJ/mol]
0	-2663.535999	0.223507	586.8	-2662.315791	0.232617	610.7
5	-2663.550745	0.208761	548.1	-2662.330214	0.218194	572.9
10	-2663.588667	0.170839	448.5	-2662.368427	0.179981	472.5
15	-2663.631447	0.128059	336.2	-2662.411811	0.136597	358.6
20	-2663.668879	0.090627	237.9	-2662.450161	0.098247	257.9
25	-2663.698310	0.061196	160.7	-2662.480847	0.067561	177.4
30	-2663.720045	0.039461	103.6	-2662.504114	0.044294	116.3
35	-2663.735293	0.024213	63.6	-2662.520845	0.027563	72.4
40	-2663.745481	0.014025	36.8	-2662.532232	0.016176	42.5
45	-2663.751960	0.007546	19.8	-2662.539562	0.008847	23.2
50	-2663.755831	0.003675	9.6	-2662.544031	0.004378	11.5
55	-2663.757998	0.001508	4.0	-2662.546576	0.001832	4.8
60	-2663.759064	0.000442	1.2	-2662.547851	0.000558	1.5
65	-2663.759473	0.000033	0.1	-2662.548342	0.000067	0.2
70	-2663.759506	0.000000	0.0	-2662.548409	0.000000	0.0
75	-2663.759366	0.000140	0.4	-2662.548288	0.000121	0.3
80	-2663.759186	0.000320	0.8	-2662.548120	0.000289	0.8
85	-2663.759060	0.000446	1.2	-2662.547992	0.000416	1.1
90	-2663.759013	0.000493	1.3	-2662.547958	0.000451	1.2

2.8. Coordination of tilted Zr_6 cluster in *d*PCN-224

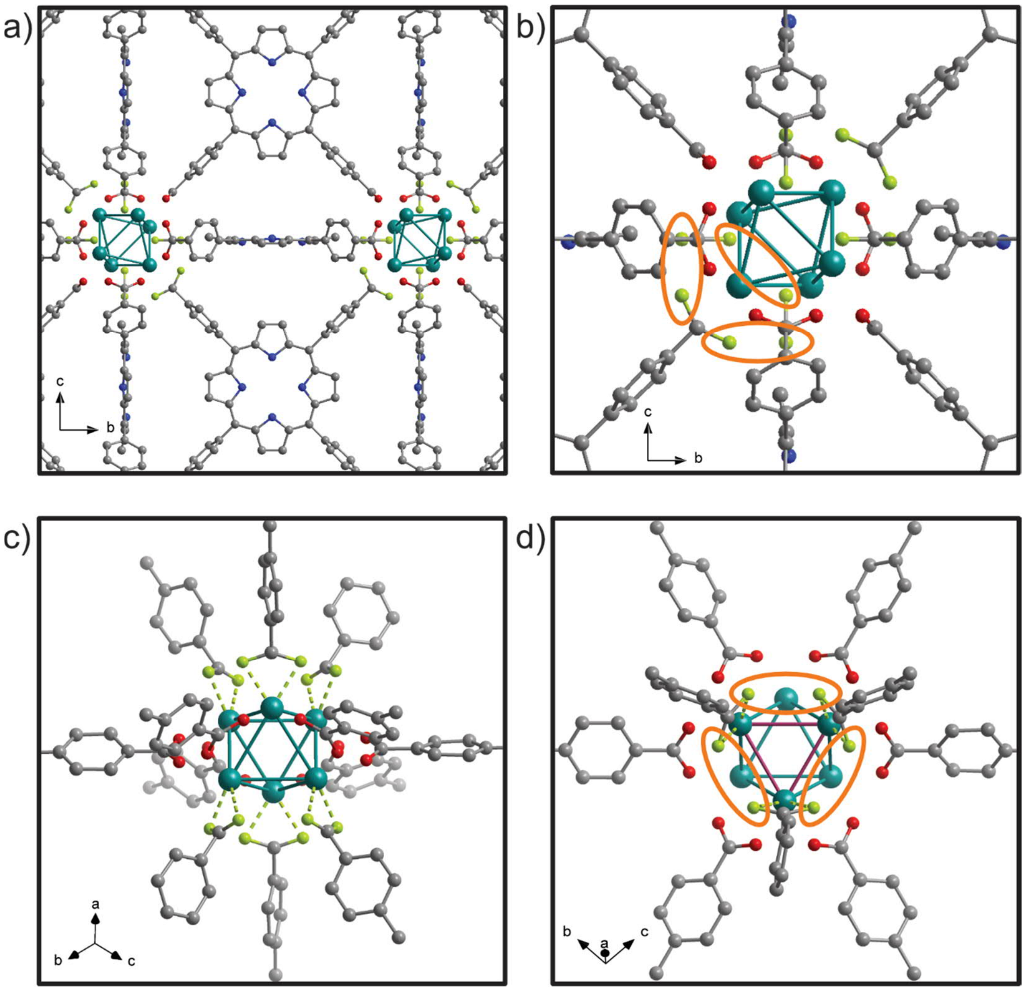

Figure S37: Coordination of the tilted Zr_6 cluster in *d*PN-224. a) Two tilted Zr_6 clusters bridged by tetrakis(4-carboxyphenyl) porphyrin in *d*PCN-224, b) zoom onto the coordination sphere of one tilted cluster with cubic coordination of the linkers, c) coordination of one tilted cluster showing six bridging linkers and six chelating linkers, and d) top-view of the chelating coordinations, showing three Zr–Zr bonds (purple) without bridging coordination. Oxygen involved in a chelating coordination are green and oxygens involved into a bridging coordination in red. Empty, additional bridging coordination sites on the tilted cluster are highlighted in orange showing that no cubic structure with more than six on-average bridging cluster-linker sites can be obtained from tilted Zr_6 cluster.

References

1. Dinnebier, R. E., Pink, M., Sieler, J. & Stephens, P. W. Novel Alkali-Metal Coordination in Phenoxides: Powder Diffraction Results on C6H5OM (M = Li, Na, K, Rb, Cs). *Inorg. Chem.* 36, 3398–3401 (1997).
2. Bruker AXS Inc. Bruker Suite, Version 2019. Bruker AXS Inc., Madison, Wi, USA. (2013).
3. Sheldrick, G. M. SADABS — Bruker AXS area detector scaling and absorption correction, version 2014/5; University of Göttingen, Göttingen, Germany. (2014).
4. Sheldrick, G. M. A short history of SHELX. *Acta Crystallogr. Sect. A Found. Crystallogr.* 64, 112–122 (2008).
5. Sheldrick, G. M. Crystal structure refinement with SHELXL. *Acta Crystallogr. Sect. C Struct. Chem.* 71, 3–8 (2015).
6. Chupas, P. J. *et al.* Applied Crystallography Rapid-acquisition pair distribution function (RA-PDF) analysis. *J. Appl. Cryst* 36, (2003).
7. Hammersley, A. P., Svensson, S. O., Hanfland, M., Fitch, A. N. & Hausermann, D. Two-dimensional detector software: From real detector to idealised image or two-theta scan. *High Press. Res.* 14, 235–248
8. Hammersley, A. P. FIT2D: A multi-purpose data reduction, analysis and visualization program. *J. Appl. Crystallogr.* 49, 646–652 (2016).
9. Juhás, P., Davis, T., Farrow, C. L. & Billinge, S. J. L. PDFgetX3 : a rapid and highly automatable program for processing powder diffraction data into total scattering pair distribution functions. *J. Appl. Crystallogr.* 46, 560–566 (2013).
10. Yang, X., Juhas, P., Farrow, C. L. & Billinge, S. J. L. xPDFsuite: an end-to-end software solution for high throughput pair distribution function transformation, visualization and analysis. 1–4 (2014).
11. Levitt, M. H. *Symmetry-Based Pulse Sequences in Magic-Angle Spinning Solid-State NMR. Encyclopedia of Magnetic Resonance* 27, (2007).
12. Kristiansen, P. E., Mitchell, D. J. & Evans, J. N. S. Double-quantum dipolar recoupling at high magic-angle spinning rates. *J. Magn. Reson.* 157, 253–266 (2002).
13. Larsen, F. H., Jakobsen, H. J., Ellis, P. D. & Nielsen, N. C. Sensitivity-enhanced quadrupolar-echo NMR of half-integer quadrupolar nuclei. Magnitudes and relative orientation of chemical shielding and quadrupolar coupling tensors. *J. Phys. Chem. A* 101, 8597–8606 (1997).
14. Carr, H. Y. & Purcell, E. M. Effects of diffusion on free precession in nuclear magnetic resonance experiments. *Phys. Rev.* 94, 630–638 (1954).
15. Meiboom, S. & Gill, D. Modified spin-echo method for measuring nuclear relaxation times. *Rev. Sci. Instrum.* 29, 688–691 (1958).
16. Pines, A., Gibby, M. G. & Waugh, J. S. Proton-enhanced nuclear induction spectroscopy. a method for high resolution nmr of dilute spins in solids. *J. Chem. Phys.* 56, 1776–1777 (1972).
17. Fung, B. M., Khitrin, A. K. & Ermolaev, K. An Improved Broadband Decoupling Sequence for Liquid Crystals and Solids. *J. Magn. Reson.* 142, 97–101 (2000).
18. Clark, S. J. *et al.* First principles methods using CASTEP. *Zeitschrift für Krist. - Cryst. Mater.* 220, 567–570 (2005).
19. Perdew, J. P., Burke, K. & Ernzerhof, M. Generalized Gradient Approximation Made Simple. *Phys. Rev. Lett.* 77, 3865–3868 (1996).
20. Monkhorst, H. J. & Pack, J. D. Special points for Brillouin-zone integrations. *Phys. Rev. B* 13, 5188–5192 (1976).
21. Tkatchenko, A. & Scheffler, M. Accurate molecular van der Waals interactions from ground-state electron density and free-atom reference data. *Phys. Rev. Lett.* (2009).
22. Charpentier, T. The PAW/GIPAW approach for computing NMR parameters: A new dimension added to NMR study of solids. *Solid State Nucl. Magn. Reson.* 40, 1–20 (2011).

23. Adamo, C. & Barone, V. Toward reliable density functional methods without adjustable parameters: The PBE0 model. *J. Chem. Phys.* 110, 6158–6170 (1999).
24. Ernzerhof, M. & Scuseria, G. E. Assessment of the Perdew–Burke–Ernzerhof exchange-correlation functional. *J. Chem. Phys.* 110, 5029–5036 (1999).
25. Grimme, S., Antony, J., Ehrlich, S. & Krieg, H. A consistent and accurate ab initio parametrization of density functional dispersion correction (DFT-D) for the 94 elements H-Pu. *J. Chem. Phys.* 132, 154104 (2010).
26. Schäfer, A., Horn, H. & Ahlrichs, R. Fully optimized contracted Gaussian basis sets for atoms Li to Kr. *J. Chem. Phys.* 97, 2571–2577 (1992).
27. TURBOMOLE V7.3 2018, a development of University of Karlsruhe and Forschungszentrum Karlsruhe GmbH, 1989-2007, TURBOMOLE GmbH, since 2007; available from http://www.turbomole.com.
28. Noh, H. *et al.* Room Temperature Synthesis of an 8-Connected Zr-Based Metal–Organic Framework for Top-Down Nanoparticle Encapsulation. *Chem. Mater.* 30, 2193–2197 (2018).
29. Kickelbick, G., Wiede, P. & Schubert, U. Variations in capping the Zr6O4(OH)4 cluster core: X-ray structure analyses of [Zr6(OH)4O4(OOC–CH=CH2)10]2(μ-OOC–CH=CH2)4 and Zr6(OH)4O4(OOCR)12(PrOH) (R=Ph, CMe=CH2). *Inorganica Chim. Acta* 284, 1–7 (1999).
30. Feng, D. *et al.* Metal-organic frameworks based on previously unknown Zr8/Hf 8 cubic clusters. *Inorg. Chem.* 52, 12661–12667 (2013).
31. Morris, W. *et al.* Synthesis, structure, and metalation of two new highly porous zirconium metal-organic frameworks. *Inorg. Chem.* 51, 6443–6445 (2012).
32. Feng, D. *et al.* Construction of ultrastable porphyrin Zr metal-organic frameworks through linker elimination. *J. Am. Chem. Soc.* 135, 17105–17110 (2013).
33. Schaate, A. *et al.* Modulated synthesis of Zr-based metal-organic frameworks: From nano to single crystals. *Chem. - A Eur. J.* 17, 6643–6651 (2011).
34. Hu, Z. *et al.* Modulator Effects on the Water-Based Synthesis of Zr/Hf Metal–Organic Frameworks: Quantitative Relationship Studies between Modulator, Synthetic Condition, and Performance. *Cryst. Growth Des.* 16, 2295–2301 (2016).
35. Stock, N. & Biswas, S. Synthesis of metal-organic frameworks (MOFs): Routes to various MOF topologies, morphologies, and composites. *Chem. Rev.* 112, 933–969 (2012).
36. Feng, D. *et al.* Zirconium-Metalloporphyrin PCN-222: Mesoporous Metal-Organic Frameworks with Ultrahigh Stability as Biomimetic Catalysts. *Angew. Chemie Int. Ed.* 51, 10307–10310 (2012).
37. Feng, D. *et al.* Construction of ultrastable porphyrin Zr metal-organic frameworks through linker elimination. *J. Am. Chem. Soc.* 135, 17105–17110 (2013).
38. Egami, T. & Billinge, S. *Underneath the Bragg peaks: structural analysis of complex materials*. (Elsevier, 2012).
39. Lorch, E. Neutron diffraction by germania, silica and radiation-damaged silica glasses. *J. Phys. C Solid State Phys.* 2, 305 (1969).
40. Soper, A. K. & Barney, E. R. On the use of modification functions when Fourier transforming total scattering data. *J. Appl. Crystallogr.* 45, 1314–1317 (2012).
41. Farrow, C. L. *et al.* PDFfit2 and PDFgui: computer programs for studying nanostructure in crystals. *J. Phys. Condens. Matter* 19, 335219 (2007).
42. Lawrence, M. C., Schneider, C. & Katz, M. J. Determining the structural stability of UiO-67 with respect to time: A solid-state NMR investigation. *Chem. Commun.* 52, 4971–4974 (2016).
43. Planas, N. *et al.* Defining the proton topology of the Zr 6 -based metal-organic framework NU-1000. *J. Phys. Chem. Lett.* 5, 3716–3723 (2014).
44. Steiner, T. The Hydrogen Bond in the Solid State. *Angew. Chemie Int. Ed.* 41, 48–76 (2002).
45. Grüninger, H. *et al.* Hidden Oceans? Unraveling the Structure of Hydrous Defects in the Earth's Deep Interior. *J. Am. Chem. Soc.* 139, 10499–10505 (2017).

46. Eckert, H., Yesinowski, J. P., Silver, L. A. & Stolper, E. M. Water in silicate glasses: Quantitation and structural studies by 1H solid echo and MAS-NMR methods. *J. Phys. Chem.* 92, 2055–2064 (1988).
47. Walther, P., Puchberger, M., Kogler, F. R., Schwarz, K. & Schubert, U. Ligand dynamics on the surface of zirconium oxo clusters. *Phys. Chem. Chem. Phys.* 11, 3640 (2009).
48. Hajek, J. *et al.* On the intrinsic dynamic nature of the rigid UiO-66 metal-organic framework. *Chem. Sci.* 9, 2723–2732 (2018).
49. Massiot, D. *et al.* 71Ga and 69Ga nuclear magnetic resonance study of β-Ga2O3: resolution of four- and six-fold coordinated Ga sites in static conditions. *Solid State Nucl. Magn. Reson.* 4, 241–248 (1995).
50. Kickelbick, G. & Schubert, U. Oxozirconium methacrylate clusters: Zr6(OH)4O4(OMc)12 and Zr4O2(OMc)12 (OMc = methacrylate). *Chem. Ber.* 130, 473–477 (1997).
