# Supplementary material for: Understanding disorder and linker deficiency in porphyrinic zirconium-based metal–organic frameworks by resolving the Zr8O6 cluster conundrum in PCN-221
Source: Nat Commun. 2021 May 25;12:3099. doi: 10.1038/s41467-021-23348-w (PMC8149457; doi:10.1038/s41467-021-23348-w)
Supplement: Supplementary file 5 — Description of Additional Supplementary Files [file 41467_2021_23348_MOESM5_ESM.docx]

Description of additional information

Title: Supplementary Data 1

Description: PCN-221/dPCN-224 structure model from refinement to single crystal x-ray diffraction data.

Title: Supplementary Data 2

Description: PCN-221/dPCN-224 structure model from refinement to powder x-ray diffraction data.

Title: Supplementary Data 3

Description: 2x2x2 supercell PCN-221/dPCN-224 structure model from refinement to powder x-ray diffraction data.
